# Multiple response optimizations on the leached-spray-dried bancha green tea towards healthy ageing

**DOI:** 10.1038/s41598-022-25644-x

**Published:** 2022-12-09

**Authors:** Vita Paramita, Nanang Masruchin, Yohanes Widodo Wirohadidjojo, Buwono Puruhito, Hermawan Dwi Ariyanto, Mohamad Endy Yulianto, Indah Hartati, Eflita Yohana, Furqon Hidayatulloh, Tris Sutrisno, Bagus Wijayanto

**Affiliations:** 1 Department of Technology Industry, Diponegoro University, Semarang, 50275 Indonesia; 2 Research Center for Biomass and Bioproducts, National Research and Innovation Agency of Indonesia (BRIN), Cibinong, Bogor, 16911 Indonesia; 3 Department of Dermatology and Venereology, Gadjah Mada University, Yogyakarta, 55281 Indonesia; 4 Department of Dermatology and Venereology, Diponegoro University, Semarang, 50275 Indonesia; 5 Department of Chemical Engineering, Wahid Hasyim University, Semarang, 50232 Indonesia; 6 Department of Mechanical Engineering, Diponegoro University, Semarang, 50275 Indonesia; 7 Kabepe Chakra, Bandung, 40971 Indonesia; 8 Metal and Wood Industry Centre, Semarang, 50111 Indonesia

**Keywords:** Biotechnology, Engineering

## Abstract

Bancha is a popular type of green tea in Japan, rich in tea polyphenols (TPs) and has a more astringent aroma with a less aromatic and strong character that complements functional foods. The blanching process is used to extract TPs and remove unwanted microorganisms, as well as inhibit phenolic oxidation. This study proposed a green tea blanching process followed by spray drying the extracts with maltodextrin. Furthermore, it is focused on maximizing the major chemical components of green tea (i.e., catechins, caffeine, and phenolic contents) based on powder particle size obtained through Multiple Response Surface Methodology optimizations. The results show that the proposed model accurately predicts leached-spray dried green tea’s total catechin and caffeine content, with a coefficient of 0.9475 and 0.8692, respectively. This process yielded composite desirability of 0.9751, while individual desirability yielded excellent results of 1.0000, 0.9188, 1.0000, and 0.9839 for catechin, caffeine, phenol content, and powder. The settings appear to yield functional results for entire responses. Due to the concerns in tropical skin nutrition applications, smaller particle size green tea can promote better adsorption than larger sizes.

## Introduction

Green tea contains many bioactive compounds dominated by polyphenols such as flavonoids, phenolic acids and tannins^1,2^, and methylxanthines such as caffeine^3^. These components confirm broad spectrum activities such as anticancer, cancer, anti-inflammation, cardiovascular disease prevention and anticarcinogens^4^. Some of the most common types of green tea in Japan are Sencha and Bancha^5^. Sencha is the first or second flush of green tea (first seasonal picking), while Bancha is the third or fourth flush (late seasonal picking)^6^. Bancha green tea has a more astringent aroma due to catechins and condensed tannins. It is much appreciated in Japan for its robust flavor and because the strong character goes well with food^7^. The process inactivating enzymes, such as polyphenol oxidase, laccase, lipoxygenase and hydro peroxidase, was performed through a blanching process. The process is also used to remove unwanted microorganisms and inhibit phenolic oxidation, known as browning. Meanwhile, browning is also known as the Maillard reaction, which results in melanoidin formation and degradation of green tea bioactive compounds^4^.

Undesired browning of food products due to enzyme activity could be effectively prevented by suppressing the activity of tyrosinase, a multifunctional glycosylated and copper-containing oxidase. Tyrosinase inhibitors are classified into natural, semi-synthetic and synthetic ones^8,9^. Natural tyrosinase inhibitors isolated from various sources such as mycological metabolites, plants, and marine algae offer myriad captivating benefits, thus promoting the research on the isolation and applications^8,10^. Quercetin, kaempferol and morin are some flavonoids showing potential inhibitory activity against tyrosinase. Moreover, tea polyphenol (TPs) such as epicatechin gallate is a natural active compound reported as a safe and promising tyrosinase activity suppressor^9^, but the inhibitory mechanism of TPs is still ambiguous. However, research on investigating the inhibition mechanism is performed. Molecular docking of tyrosinase and TPs was performed, and the dominant role in the binding of ECG to tyrosinase is played by hydrophobic and hydrogen bonding forces^9,10^. It was also stated that poly-condensate aldehyde of catechin reduces the tyrosinase activity by chelating the active sites^10^. Furthermore, through a cresolase-like pathway, epicatechin gallate (ECG) and catechin (C) make tyrosinase irreversibly inactivated due to their catechol group (ring B) being catalyzed by tyrosinase. Epigallocatechin gallate (EGCG) inhibits the activity of tyrosinase by competing with or delaying the oxidation of substrate^2^.

Epigallocatechin gallate, epicatechin, epigallocatechin and epicatechin gallate are the primary polyphenols present in green tea^11^. The most effective attribute in scavenging the alkyl peroxyl radical was EGCG, which performed 10 times better than vitamin C and -carotene^12,13^. Caffeine was also reported as a significant tyrosinase inhibitor, and microencapsulation using a spray dryer is important in the pharmaceutical and food industries. This process is suitable for sensitive and volatile materials and can increase product stability^14^. Therefore, TPs of bancha were separated by the applied blanching process and followed by spray drying of the green tea extracts. Maltodextrin was utilized as wall material in the spray dying, focusing on maximizing the major chemical components of green tea on the powder particle size obtained by using multiple response surface methodology optimizations.

## Methods

### Materials

The dried bancha green tea leave was obtained from one Indonesian Tea Board member, PT. Kabepe Chakra (Bandung, Indonesia). The production process by Kapebe Cakra was in line with the Indonesian National Standard (SNI no 3945:2016), Ethical Tea Partnership, Rainforest Alliance and Halal Assurance System. The chemicals used during the analysis were Folin–Ciocâlteu reagent (FCR), carbon tetrachloride (CCl_4_), chloroform (CHCl_3_), and sodium carbonate (Na_2_CO_3_), which were all purchased from Merck & Co., Inc. (New Jersey, US). Furthermore, Sigma-Aldrich Fine Chemicals supplied the gallic acid (Missouri, US). The experimental research on plants (tea) complied with the relevant institutional, national, and international guidelines and legislation. Plants of tea applied in this research did not involving species at risk of extinction.

### General procedure

Fifty grams of dried bancha green tea leave were weighed and leached regarding the designed variables of temperature, leave-to-water ratio and time. Response Surface Methodology was applied to obtain the optimal condition during leaching. Furthermore, the leached bancha tea was spray dried at 30 °C and 80 °C of liquid and hot air inlet temperature. The obtained powder analyzed the major chemical contents of catechin, caffeine and phenol by conducting duplicate absorbance measuring of UV Vis spectrophotometer (GENESYS™ 10 Series, Massachusetts, US). The powder particle size was analyzed using a particle size analyzer (Horiba LA-960, Japan). Furthermore, the experimental research on bancha tea conformed to the Good Agriculture Practices/GAP on Tea^15^ and Compendium of Guidelines for Tea^16^.

### Determination of caffeine content

Ten millilitres of distilled water were added to 5 mg of sample, followed by 1 mL of 20% Na_2_CO_3_ solution and 20 mL of CCl_4_. The extraction of caffeine was obtained by forming a non-polar layer of CCl_4_. Subsequently, CCl_4_ (20 mL) was added to the mixture solution and separated by forming a non-polar layer of CCl_4_. This stage was repeated until it reached 50 mL of solvent. The separated solvent was then analyzed for the caffeine content using the UV–Vis spectrophotometer (GENESYS™ 10 Series, Massachusetts, US) at 270 nm. This procedure was employed in duplicate measurements^17^.

### Determination of total catechin content

Fifty milligrams of powder were dissolved in 40 mL of distilled water. Furthermore, 40 mL of CHCl_3_ was employed for washing to remove the non-polar impurities, including caffeine and pigments. This washing procedure was repeated four times, following the extract’s absorbance analysis at 274 nm by conducting UV–Vis Spectrophotometry (GENESYS™ 10 Series, Massachusetts, US)^13^.

### Determination of total phenol content

The FCR method was applied for total phenol content determination. One milligram of powder was dissolved on 1 mL of distilled water and mixed with a gallic acid standard of 100 µg/mL. This was followed by adding 5 mL of distilled water, 0.5 mL of FCR, 1.5 mL of 20% Na_2_CO_3_ and distilled water until it reached a total volume of 10 mL. After 2 h of incubation and the dark blue color of the solution was obtained, the absorbance was determined using a UV–Vis Spectrophotometer (GENESYS™ 10 Series, Massachusetts, US) at 750 nm^18,19^.

### Determination of powder particle size

In dry form, the particles obtained were analyzed using a particle size analyzer (Horiba LA-960, Japan). Twenty grams of sample was fed into a dry cell chamber in the auto mode set up with 97–98% transmittance. The particles were air scattered with purified air at 0.3 MPa, and the measurement was conducted in three replications^20^.

### Surface structure determination

The surface morphology of samples was observed under scanning electron microscopy (SEM) (Thermo Scientific Quattro S, Germany) without any coating. A pinch of sample powder was placed on the double taped stubs, and then the SEM was operated at a high vacuum with a voltage of 2 kV at the magnification of 250 × , 500 × , and 1500 × .

### Multiple response surface methodology

The alpha’s central composite design (CCD) for orthogonality is used to analyze the multiple response surface experiments (Minitab 19 Statistical Software, Pennsylvania, US). The independent variables of the leaching process of bancha green tea were temperature (X_1_), leave-to-water ratio (X_2_) and leaching time (X_3_). Each optimized variable was coded at five levels, namely − α, − 1, 0, + 1 and + α, regarding the range of leaching process of bancha green tea, as shown in Table 1. The multiple responses obtained were caffeine content (FC), total catechin content (TC), total phenolic content (PC) and powder particle size (PS).

**Table 1:** Central composite design for multi-response optimization of spray-dried bancha green tea.

Independent variables	Coded variables levels				
	− α	− 1	0	+ 1	+ α
Temperature (°C)	71.59	75.00	80.00	85.00	88.41
Leave-to-water ratio (–)	0.066	0.100	0.150	0.200	0.234
Time (min)	3.296	5.000	7.500	10.00	11.70

## Results and discussion

### Effect of extraction parameter on the major chemical components and physical properties of bancha leached-spray dried green tea

The leaching process of bancha was conducted by using a central composite design with alpha for the orthogonality. The parameters were leaching temperature, leave-to-water ratio, and leaching time. A set of 16 experiments and the observed responses of spray-dried powder of the extract obtained are tabulated in Table 2. Green tea extractions for functional foods are commonly conducted using water. It is cost-effective, in batch or continuous processes on a commercial scale, to remove undesirable constituents and centralize bioactive compounds^21,22^. The extraction efficiency is proportional to the solvent-to-water ratio and is heavily influenced by the polarity of the constituent compounds and the solvent type^23^.

**Table 2:** Experimental design matrix of leaching and observed responses of spray-dried powder of extract bancha green tea.

Run	Experimental design matrix of leaching			Observed responses			
	X_1_	X_2_	X_3_	Y_1_	Y_2_	Y_3_	Y_4_
	Temperature (°C)	Leave-to-water ratio (gr/mL)	Time (min)	Total catechin content (mg/mL)	Caffeine content (mg/mL)	Total phenolic content (mg gallic acid equivalent/g dry material)	Powder Particle Size (μm)
1	75.0	0.10	5.00	0.737 ± 0.016	24.17 ± 0.534	0.039 ± 0.001	347.9 ± 37.125
2	85.0	0.10	5.00	1.411 ± 0.037	36.58 ± 0.467	0.047 ± 0.001	564.5 ± 38.435
3	75.0	0.20	5.00	1.578 ± 0.010	54.45 ± 0.267	0.085 ± 0.002	432.9 ± 39.804
4	85.0	0.20	5.00	2.060 ± 0.063	63.51 ± 0.800	0.024 ± 0.000	541.7 ± 34.822
5	75.0	0.10	10.0	1.715 ± 0.016	31.15 ± 0.267	0.052 ± 0.000	543.5 ± 36.956
6	85.0	0.10	10.0	2.097 ± 0.042	56.81 ± 0.667	0.076 ± 0.000	405.5 ± 3.882
7	75.0	0.20	10.0	1.667 ± 0.021	47.24 ± 0.334	0.063 ± 0.001	336.9 ± 46.849
8	85.0	0.20	10.0	1.671 ± 0.016	37.94 ± 0.534	0.040 ± 0.001	339.3 ± 19.500
9	73.6	0.15	7.50	1.182 ± 0.037	31.53 ± 1.201	0.026 ± 0.001	355.6 ± 22.821
10	86.4	0.15	7.50	2.279 ± 0.026	58.89 ± 1.067	0.050 ± 0.000	435.3 ± 27.631
11	80.0	0.09	7.50	0.885 ± 0.026	33.13 ± 0.934	0.038 ± 0.001	353.2 ± 23.723
12	80.0	0.21	7.50	1.675 ± 0.052	60.92 ± 0.867	0.086 ± 0.001	738.4 ± 8.475
13	80.0	0.15	4.28	0.830 ± 0.031	49.92 ± 0.534	0.063 ± 0.001	358.4 ± 39.543
14	80.0	0.15	10.7	0.874 ± 0.021	47.47 ± 0.800	0.031 ± 0.000	467.5 ± 28.763
15	80.0	0.15	7.50	0.833 ± 0.047	46.43 ± 0.133	0.057 ± 0.001	548.0 ± 18.957
16	80.0	0.15	7.50	0.841 ± 0.026	42.19 ± 0.667	0.043 ± 0.001	443.0 ± 14.608

Leaching temperature and time are critical in the major chemical components, varying efficiency depending on the compound^24^. The experimental outcomes show that these variables impacted the content of caffeine, catechin, and phenolic. This is also supported by the research^25^ that optimized leaching conditions from green tea bags and total phenolic content increase with temperature and time. Another study conducted by^26^ where caffeine was water leached from Turkish tea, found that leaching temperature affects the caffeine content, with the maximum yield obtained at its boiling point. Heat makes tea cell walls more permeable to solvent penetration, thus increasing the components’ solubility and diffusion coefficients. However, applying higher extraction temperature can also impact solvent viscosity reduction and catechins’ degradation^27–29^. The ratio of leaves to water significantly affects the catechin content. Similarly, the higher the solvent used for extraction, the more catechin is extracted^30,31^. To visualize the relationship amongst the response and experimental results of the independent variables for the leaching condition, a response surface profile based on the quadratic polynomial model equation was generated, as seen in Table 4.

### Effect of particle size on the catechins contents, caffeine contents and phenol content

**Figure 1:**
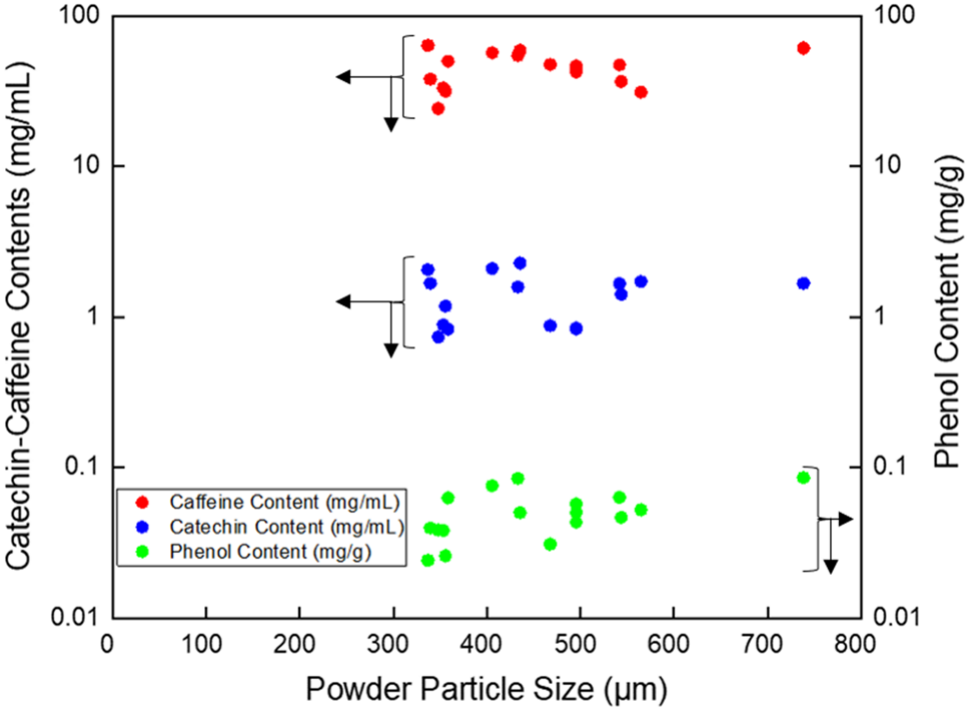
Distribution of major chemical contents of bancha green tea regarding the powder particle size.

**Figure 2:**
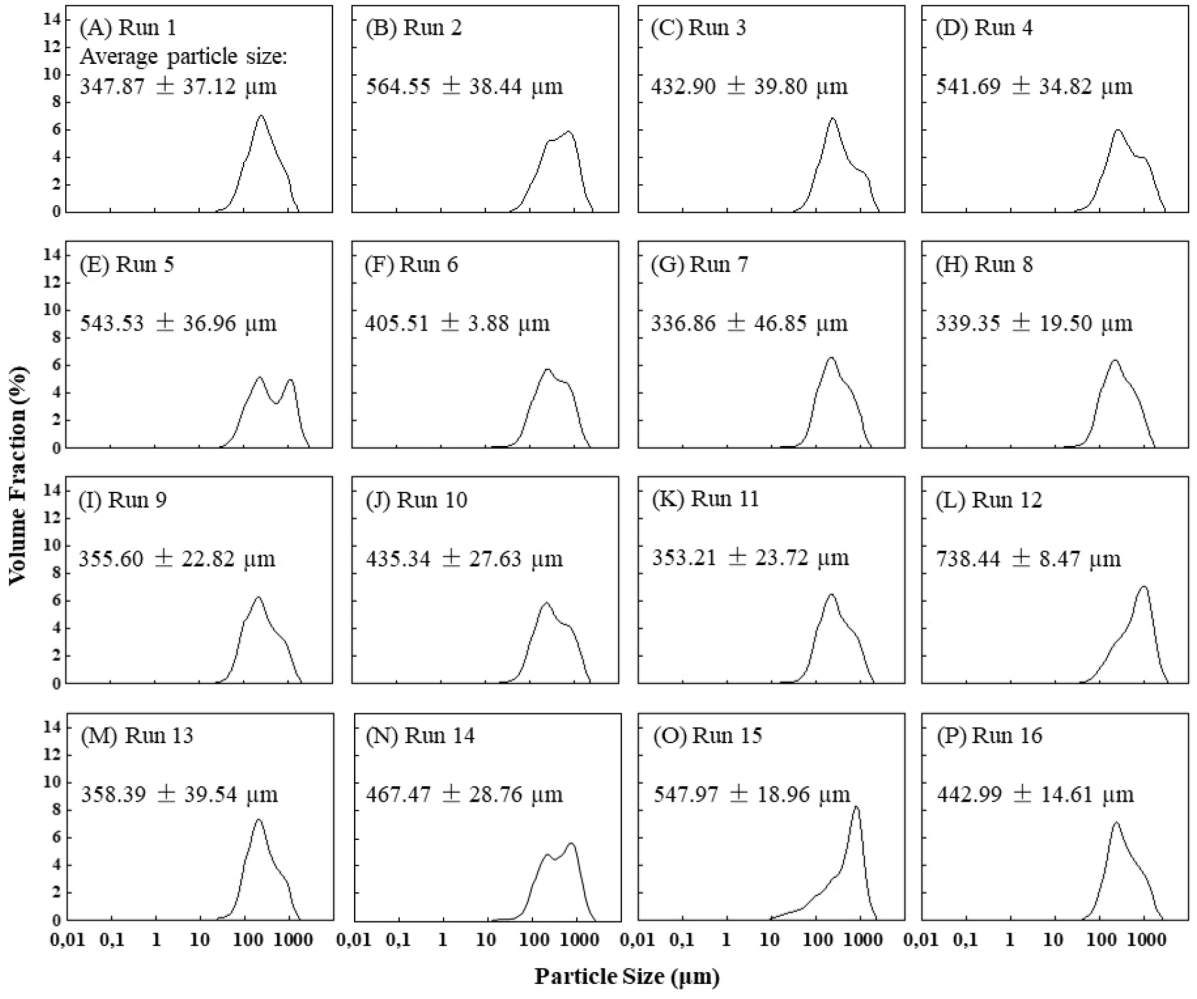
The powder particle size of the run parameters: (**A**) Run 1; (**B**) Run 2; (**C**) Run 3; (**D**) Run 4; (**E**) Run 5; (**F**) Run 6; (**G**) Run 7; (**H**) Run 8; (**I**) Run 9; (**J**) Run 10; (**K**) Run 11; (**L**) Run 12; (**M**) Run 12; (**N**) Run 14; (**O**) Run 15; (**P**) Run 16.

The particle size of bancha green tea powder was determined by an analyzer (Fig. 2A–P). The distribution of major chemical contents is separated into caffeine, catechin, and phenol. As can be seen in Fig. 1 that dried bancha green tea performed the highest caffeine compared to the catechin content and provided the lowest minimum content for all variables. The particle size was completely affected by the air temperature, proofed in larger particles in the inlet air temperature at 132 °C than 180 °C^32^. Increasing the drying air temperature results in increased particle size due to ballooning or puffing^33^. Caffeine content was found as the highest component in bancha green tea, with a particle size of 337 μm (Fig. 2G) in the amount of 63.51 g/mL. Meanwhile, catechin content was the second-place major chemical content of bancha green tea with a particle size of 435 μm (Fig. 2J) in the amount of 2.279 g/mL total catechin content. Particle size significantly affected total catechin concentration^34^. The concentration of caffeine and total catechins were greatly affected by the particle size of green tea. However, as particle size increased from 433 μm (Fig. 2C) to 467 μm (Fig. 2N), all the major content levels decreased, especially catechin (0.875 g/mL). It is supported by the report of^35^ that catechin contents decreased as particle size increased because of the contact surface between O_2_ and particles resulting in catechin oxidation. The tea powder’s particle size influences the solute’s mass transfer and the total surface area for contact between the solvent and the sample^34^.

### Parameters effect on catechins, caffeine and phenolic contents

The optimization studies of biomolecule component extraction of catechin^36,37^, caffeine^38,39^ and phenolic contents^40,41^ were conducted and observed using the response surface methodology (RSM). Table 3 shows the variance analysis of caffeine, catechin, phenolic contents and powder particle size against temperature, leaves-to-water, and time. The error on the degree of freedom (DF) was found at 10 consisting of 5 lack-of-fit and 5 pure errors. This indicates the independent information which can estimate the coefficients, and the error of DF is directly proportional to the precision of the coefficient verified in regression. Applied Fisher’s F test evaluated the model’s regression coefficient’s significance. The F0, the computed value of F, of the model for catechin, caffeine, phenolic, and powder particle size were 20.05, 1.67, 7.38 and 1.18, respectively. The computed value of F of catechin and phenolic content exceed 4.96 (the value of F0.05,1,10), indicating the significance of both models. Furthermore, Table 3 serves information of terms in four models that have significant effect on the response. The information is in form of its p-value indicated with star sign next to the adjusted sum of squares. The p-value less than 0.05 represent that the terms of model have a significant effect on the response. In case of catechin content model, the linear terms which include temperature, leave-water ratio and time were statistically significant. The quadratic term of temperature × temperature as well as leave-water ratio × leave water ratio was also statistically significant. It was also found that 2- way interaction of leave-water ratio × time is also significant for catechin content model. The significant effect of linier term of temperature, time and leave – water ratio on catechin separation from peanut red skin was also reported^42^. Variance analysis also shows that the 2-way interaction of temperature × leave-water ratio was the only term statistically significant on caffeine content model. The liner term of temperature, leave-water ratio and time were statistically significant. Furthermore, 2- way interaction of temperature × leave-water ratio and leave-water ratio × time were statistically significant on phenolic content model. The significance of temperature and sample-solvent ratio on phenolic substance extraction was also reported^43^. They mention that sample—solvent ratio was the most significant single factor in influencing the antioxidant capacity of the phenolic extract of ground ivy obtained from microwave assisted extraction process.

**Table 3:** Performance of variance analysis for catechin, caffeine, phenolic contents and powder particle size against temperature, leave-to-water and time of leaching conditions.

Source	DF	Adjusted sums of squares (Adj SS)			
		Catechin content	Caffeine content	Phenolic content	Powder particle size
Model	9	4.76947*	1846.76*	0.003292	97,661
Linear	3	1.31473*	1197.40*	0.000358	19,074
Temp (°C)	1	0.77132*	471.61*	0.000039	2238
L/W (–)	1	0.36487*	719.07*	0.000317	7190
Time (min)	1	0.17853*	6.73	0.000002	9646
Square	3	2.85735*	12.73	0.000614	37,860
Temp (°C) × Temp (°C)	1	1.80723*	9.36	0.000202	17,902
L/W (–) × L/W (–)	1	0.42421*	0.07	0.000507	9,522
Time (min) × Time (min)	1	0.00006	5.35	0.000000	11,434
2-Way Interaction	3	0.59739*	636.62*	0.002320	40,728
Temp (°C) × L/W (–)	1	0.04073	183.38*	0.001678*	14,030
Temp (°C) × Time (min)	1	0.07429	3.24	0.000344	26,565
L/W (–) × Time (min)	1	0.48237*	450.00*	0.000299	133
Error	10	0.26425	277.83	0.002188	91,285
Lack-of-Fit	5	0.26425	277.83	0.002188	91,285
Pure Error	5	0.00000	0.00	0.000000	0
Total	19	5.03372	2124.59	0.005480	188,947

*DF* Degree of freedom.

* = < 0.05 of *p* value.

Table 4 presents the surface responses of the quadratic polynomial model equation, the standard error of the regression (S) and the coefficient of determination (R^2^). The coefficient of determination (R-square) was obtained at 0.9475, 0.8692, 0.6007 and 0.5169 for contents of catechin, caffeine, total phenolic and powder particle size. The R-square (in the range of 0–1) of a model should be larger than 0.8 to be a good predictive model^44^. Therefore, the R-square significantly fits the catechin and caffeine content models. These prediction values were strengthened by the standard error of the regression (0.1626, 5.2709, 0.0148 and 95.5433 of each response variable). The closer fitting line indicators and the smaller standard error values are preferable.

**Table 4:** Model equations of the responses.

Response	Quadratic polynomial model equations	Standard error	R^2^
Total catechin content (TC)	122.7 − 3.222 X_1_ + 10.50 X_2_ + 0.969 X_3_ + 0.021 X_1_^2^ + 102.2 X_2_^2^ − 0.0005 X_3_^2^ − 0.285 X_1_X_2_ − 0.0077 X_1_X_3_ − 1.964 X_2_X_3_	0.1626	0.9475
Caffeine content (FC)	− 708.0 + 12.20 X_1_ + 2154 X_2_ + 10.60 X_3_ − 0.0480 X_1_^2^ − 42.00 X_2_^2^ + 0.145 X_3_^2^ − 19.15 X_1_X_2_ − 0.051 X_1_X_3_ − 60.0 X_2_X_3_	5.2709	0.8692
Total phenolic content (PC)	− 1.720 + 0.040 X_1_ + 4.050 X_2_ − 0.0342 X_3_ − 0.0002 X_1_^2^ + 3.530 X_2_^2^ − 0.00004 X_3_^2^ − 0.0579 X_1_X_2_ + 0.0005 X_1_X_3_ − 0.0489 X_2_X_3_	0.0148	0.6007
Powder particle size (PS)	− 17,653 + 393 X_1_ + 9068 X_2_ + 476 X_3_ − 2.10 X_1_^2^ + 15,308 X_2_^2^ − 6.71 X_3_^2^ − 168 X_1_X_2_ − 4.61 X_1_X_3_ + 33 X_2_X_3_	95.543	0.5169

**Figure 3:**
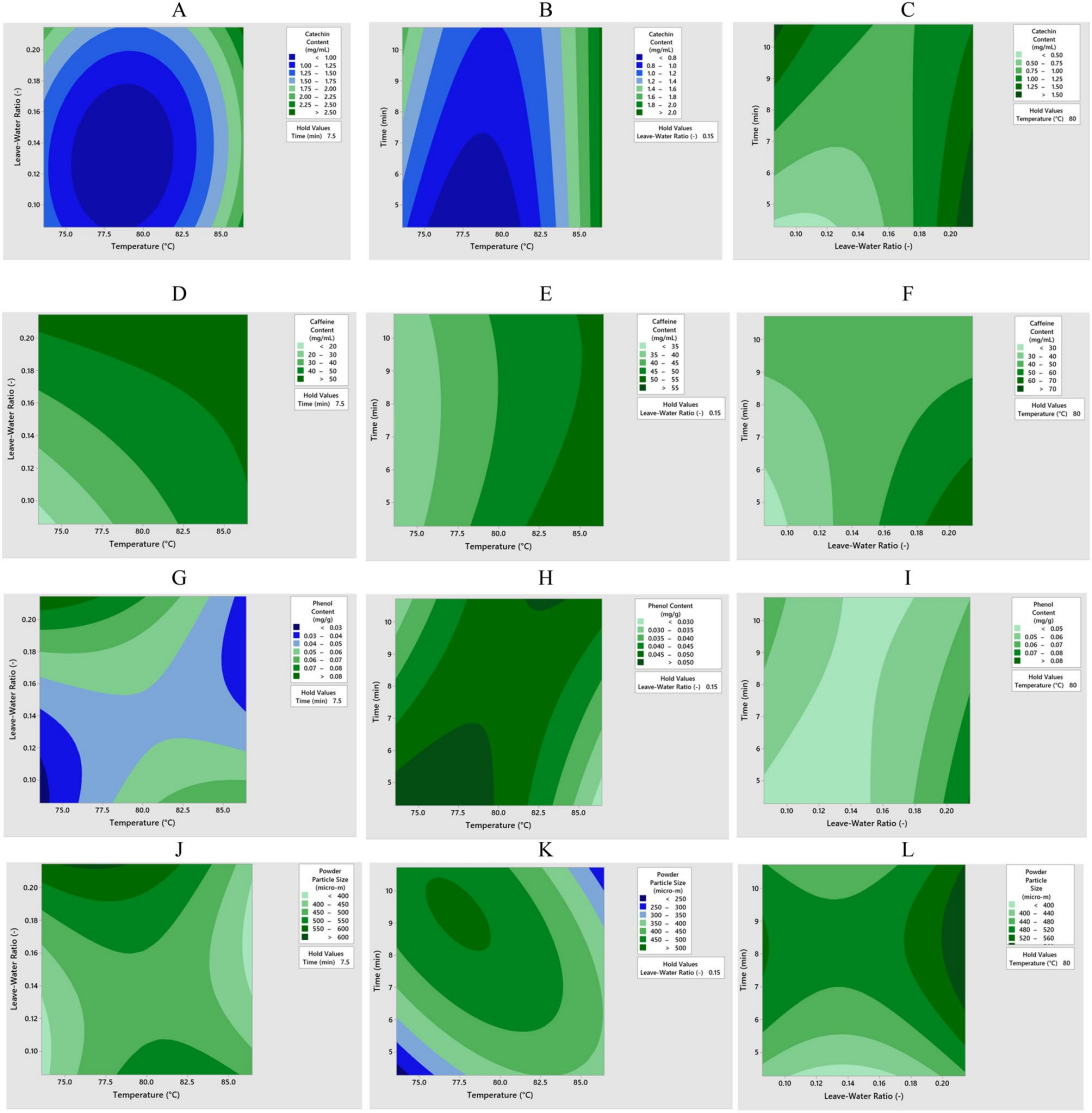
Response surface and profile of total catechin content (**A**–**C**), caffeine content (**D**–**F**), total phenolic content (**G**–**I**) and powder particle size (**J**–**L**) regarding temperature-L/W ratio at 7.5 min of leaching time (**A**, **D**, **G**, **J**), temperature–time at L/W ratio 0.15 (**B**, **E**, **H**, **K**), L/W ratio-time at 80 °C (**C**, **F**, **I**, **L**).

The response variables and the interaction effect on the contour plots are shown in Fig. 3A–L. The interactive effect of catechin content was regarding the leaching temperature and leave-to-water ratio at 7.5 min of leaching time, as represented in Fig. 3A. The maximum catechin content was obtained at the highest leaching temperature and time. Regarding the leaching temperature and time at 0.15 of leave-to-water ratio, Fig. 3B shows the maximum catechin content. It was obtained at the highest leaching temperature, regardless of the time. Figure 3C depicts the plot of catechin content regarding the leaching time vs. leave-to-water ratio at 80 °C of leaching temperature. The maximum catechin was achieved at the lowest leave-to-water ratio with the longest leaching time. The minimum bancha green tea needed a longer time to diffuse in the water as a solvent. Increasing the bancha green tea by more than 0.2 of the leave-to-water ratio provided the maximum catechin content at a faster time up to 7 min. The high temperature could increase the permeability of green tea cell walls for solvent and constituent^27^. However, the high temperature could degrade these contents because epimerization can change their structure^45^. Due to water efficiency and cost-effectiveness, a low leaves-to-water ratio is desirable for extracting catechins^46^. The longer the leaching duration, the greater the chance of catechin thermal decomposition into other chemicals^27^. The decrease of catechin content indicates this after 7 min of leaching time.

Figure 3D illustrates the caffeine content concerning leave-to-water ratio and leaching temperature at 7.5 min of leaching time. The total catechin content was obtained at the maximum level of leave-to-water ratio and temperature. Regarding the time and temperature of leaching at a 0.15 leave-to-water ratio, the maximum catechin content was obtained at the maximum temperature, disregarding the leaching time (Fig. 3E). From the other point of view, Fig. 3F represents the leaching temperature at 80 °C and found the highest catechin content at the highest leave-to-water ratio regarding shorter time than 6 min.

The maximum phenol content was observed at the low temperature (< 77 °C) with regards to the high leave-water ratio (> 0.21) at a hold time value of 7.5 min (Fig. 3G). Figure 3H indicates the phenol content (0.05 mg/g) in the range of 5.4–6.7 min with regards to a lower temperature than 79.5 °C. Meanwhile, Fig. 3I depicts the phenol content at 80 °C, showing that the maximum phenol content (0.08 mg/g) was found at a leave-water ratio of 0.2 during leaching time for 7 min. The longer leaching time than 7.5 min decreases the phenol content due to thermal degradation.

Subsequently, the particle size (< 400 μm) was obtained at a higher temperature than 87 °C in the range of 0.13–0.20 of leave-water ratio for 7.5 min (Fig. 3J). At a 0.15 leave-water ratio, the minimum particle size of 250 μm was determined at leaching conditions of 72.5 °C and 4 min (Fig. 3K). Figure 3L depicts the minimum powder particle size (< 400 μm) on the leaching time (< 4.5 min) and the range of leave-water ratio at 0.10–0.17.

According to the findings, the temperature is directly proportional to the caffeine content in the extracted green tea^47,48^. Raising the temperature to 95 °C reduces the total catechins content, and this explains why it undergoes thermal degradation and epimerization^48,49^. The temperature was raised above 90 °C, decreasing total phenolic content^50^.

### Response of optimization of phenolic, catechins, caffeine contents and powder particle size

The optimization response of bancha green tea components and powder particle size obtained during spray-dried was defined. The observation comprises the maximum values of catechin, caffeine, and phenolic contents on the minimum value of powder particle size (Table 5). The prediction of the multiple responses obtained the setting variable at 86.4 °C, 0.0895 of leaves-to water ratio and 10.72 min of leaching time for the fit solution (2.699 mg/mL, 60.31 mg/mL, 0.0862 mg/g and 343 μm for catechin, caffeine, phenolic contents and powder particle size, respectively).

**Table 5:** Solution of multiple response prediction.

Parameters	Verification analyzed	Solution		Individual desirability	Composite desirability
		Fit	SE fit		
**Variable setting**					
Temperature (°C)	86.4	86.4			
Leaves-water ratio (–)	0.0895411	0.0895411			
Time (min)	10.7180	10.7180			
**Responses**					
TC (mg/mL; max)	15.296 ± 3.381	2.699	0.214	1.0000	0.9751
FC (mg/mL; max)	276.245 ± 0.800	60.31	6.94	0.9188	
PC (mg/g; max)	230.453 ± 23.481	0.0862	0.0195	1.0000	
PS (μm; min)	214.14 ± 4.62	343	126	0.9839	

*SE* Standard error, *TC* Total catechin content, *FC* Caffeine content, *PC* Total phenolic content, *PS* Powder particle size.

**Figure 4:**
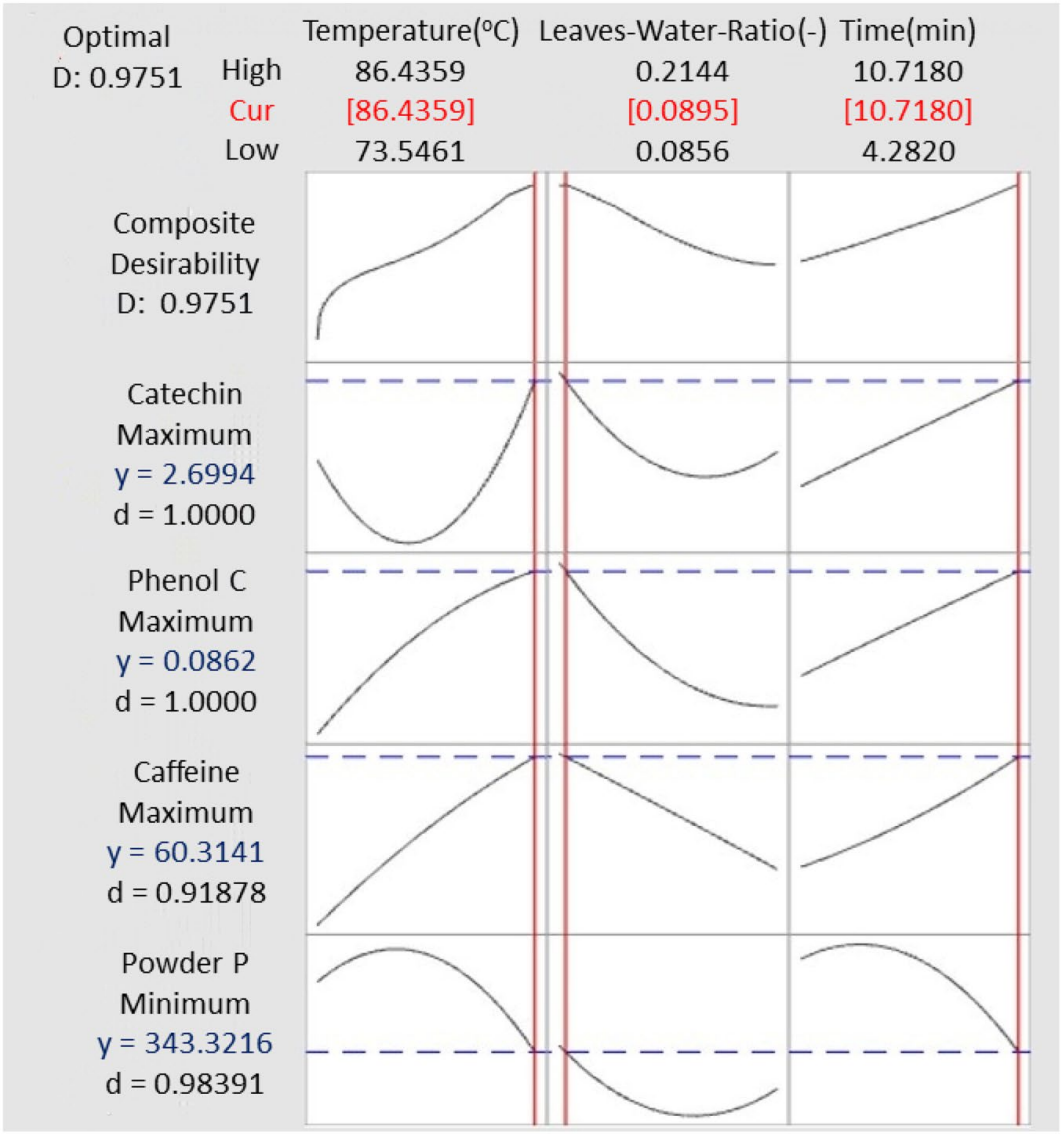
Multiple response prediction on the maximum value of catechin, caffeine and total phenolic contents regarding the minimum value of powder particle size.

Figure 4 shows the individual and composite desirability of the response parameter. The individual and composite desirability reported how much the variable optimized the single and the entire response. The desirability approach transforms each predicted response’s measured properties into a dimensionless value of d. The scale of the desirability function ranges between 0–1, and a value of zero (d = 0) implies that the response is completely unacceptable. In contrast, when the desirability value is unity, the response is exactly of the targeted value^51^. The desirability values were tabulated in Table 5, and the composite desirability was found to be 0.9751. The individual desirability was found at 1.000, 0.9188, 1.000, and 0.9839 for each catechin, caffeine, phenolic contents and powder particle size response.

**Figure 5:**
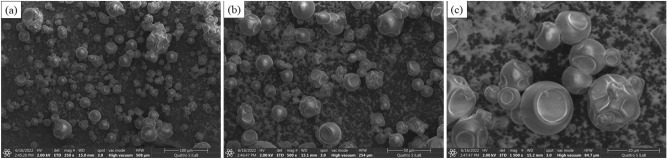
SEM images of microcapsules on 86.4 °C leaching temperature, 0.09 of leaves-water ratio and 10.72 min. Magnification: 250 × (**a**); 500 × (**b**); 1500 × (**c**).

In conclusion, the variables obtained favorable results for all responses and are more effective at maximizing the catechin and phenolic contents yield. Figure 5 presents SEM micrographs of the predicted setting variables. The powder showed dents surface structure without cracks, confirming the extended film formulation during the early phase under low temperature drying^52,53^.

## Conclusion

The proposed model satisfies the leached-spray dried green tea prediction, consisting of 0.9475 and 0.8692 total catechin and caffeine content on the coefficient of determination. The process provided the composite of 0.9751, and the individual desirability provided excellent results of 1.0000, 0.9188, 1.0000 and 0.9839 for catechin, caffeine, phenol contents and powder particle size. Therefore, the settings appear to obtain the functioning results for whole responses. The individual desirability indicates that the settings are shown potentially to maximize catechin and phenol contents (1.000) by minimizing the powder particle size (0.9839) than maximizing caffeine content (0.9188). Since powder particle size plays the main role in topical skin nutrition, it can promote better adsorption of the main component of green tea.

## Supplementary Information

Supplementary Information.

## Data Availability

The datasets used and analyzed during the current study are available from the corresponding author on reasonable request.
