# Supplementary material for: Multiple response optimizations on the leached-spray-dried bancha green tea towards healthy ageing
Source: Sci Rep. 2022 Dec 9;12:21347. doi: 10.1038/s41598-022-25644-x (PMC9734194; doi:10.1038/s41598-022-25644-x)
Supplement: Supplementary file 1 — Supplementary Information. [file 41598_2022_25644_MOESM1_ESM.pdf]

20210208,MINITAB.MWX

Response Optimization: Catechin Content (mg/mL), Phenol Content (mg/g), Caffeine Content (mg/mL), Powder Particle Size (micro-m)

Parameters

Response	Goal	Lower	Target	Upper	Weight	Importance
Catechin Content (mg/mL)	Maximum	0.7368	2.279		1	1
Phenol Content (mg/g)	Maximum	0.0242	0.086		1	1
Caffeine Content (mg/mL)	Maximum	24.1698	63.509		1	1
Powder Particle Size (micro-m)	Minimum		336.861	738.435	1	1

Solution

Solution	Temperature (°C)	Leave-Water Ratio (-)	Time (min)	Catechin Content (mg/mL) Fit	Phenol Content (mg/g) Fit	Caffeine Content (mg/mL) Fit	Powder Particle Size (micro-m) Fit
1	86.4359	0.0895411	10.7180	2.69940	0.0862410	60.3141	343.322

Solution	Composite Desirability
1	0.975083

Multiple Response Prediction

Variable	Setting
Temperature (°C)	86.4359
Leave-Water Ratio (-)	0.0895411
Time (min)	10.718

Response	Fit	SE Fit	95% CI	95% PI
Catechin Content (mg/mL)	2.699	0.214	(2.223, 3.176)	(2.101, 3.298)
Phenol Content (mg/g)	0.0862	0.0195	(0.0429, 0.1296)	(0.0318, 0.1407)
Caffeine Content (mg/mL)	60.31	6.94	(44.86, 75.77)	(40.90, 79.73)
Powder Particle Size (micro-m)	343	126	(63, 624)	(-9, 695)

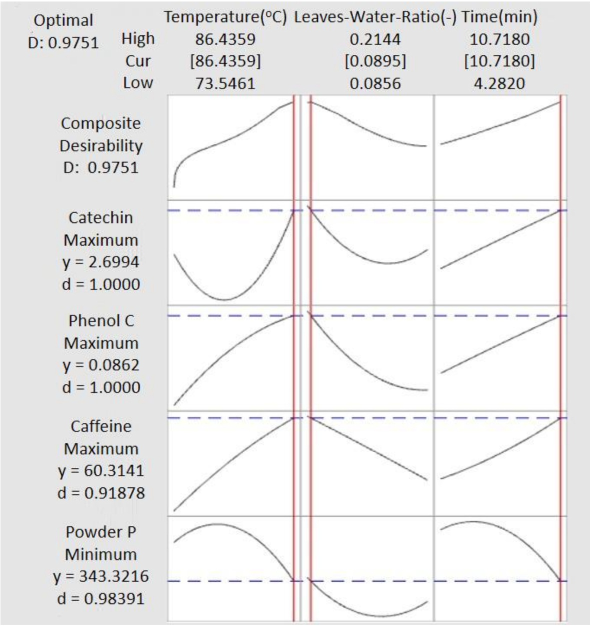

20210208,MINITAB.MWX

Contour Plots of Catechin Content (mg/mL)

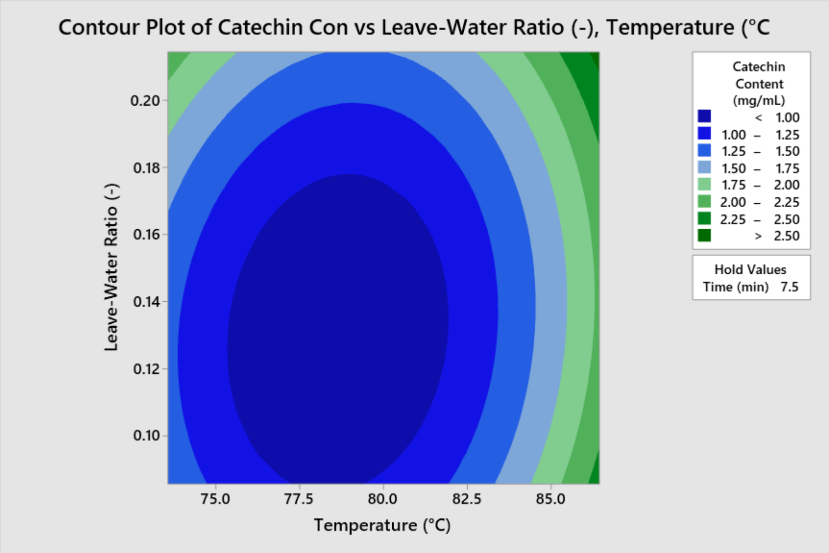

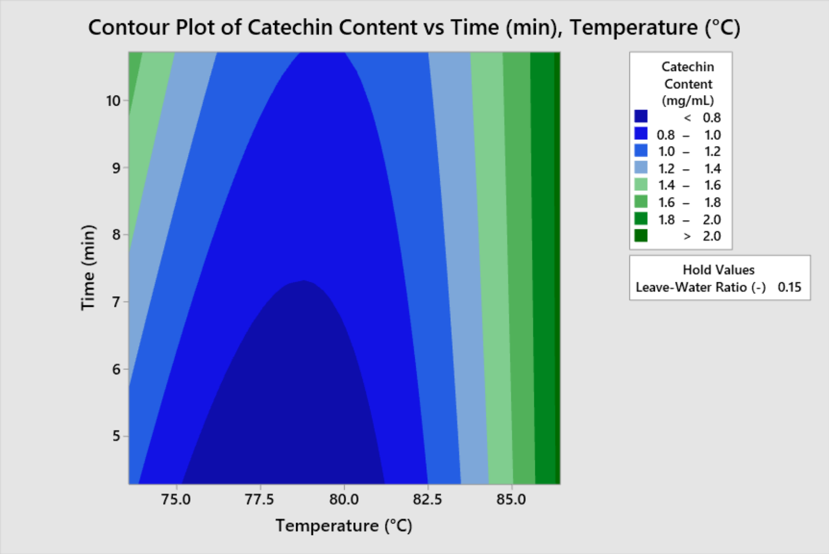

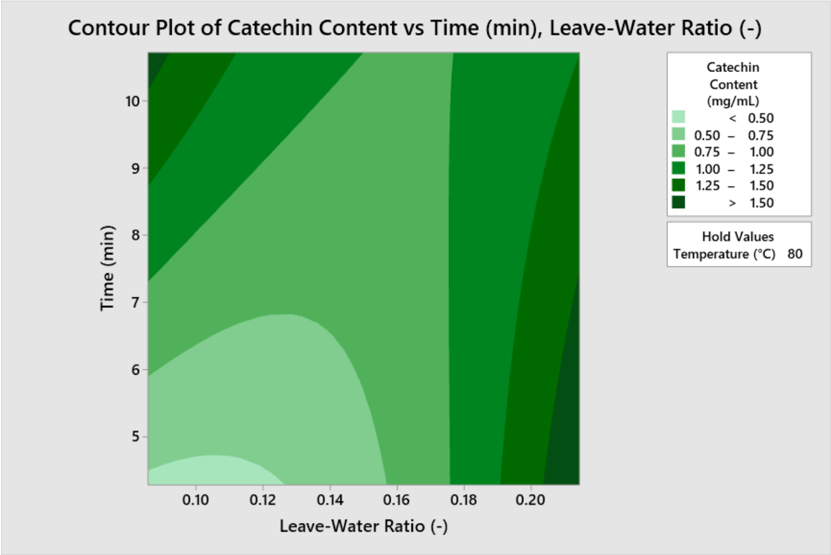

20210208,MINITAB.MWX

Surface Plots of Catechin Content (mg/mL)

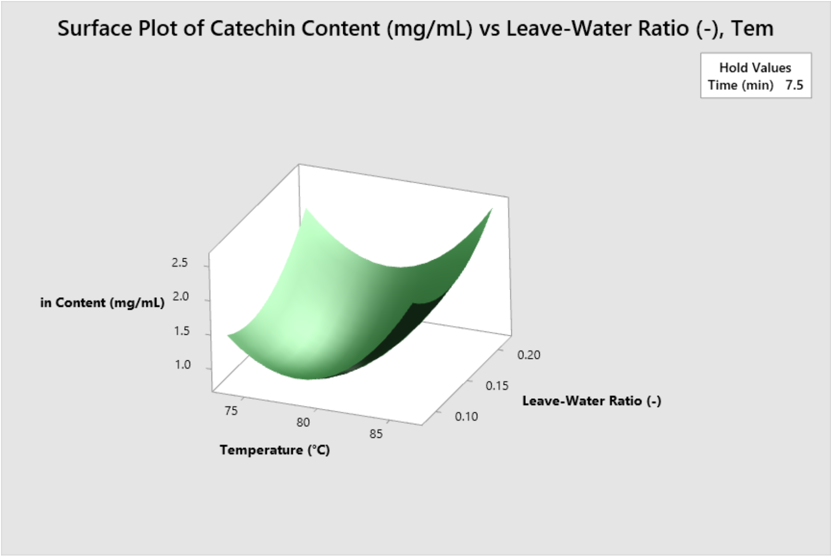

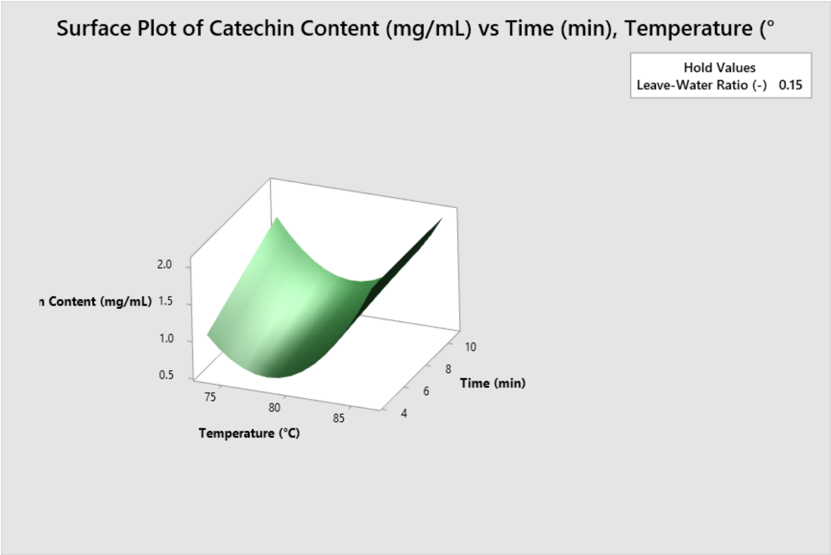

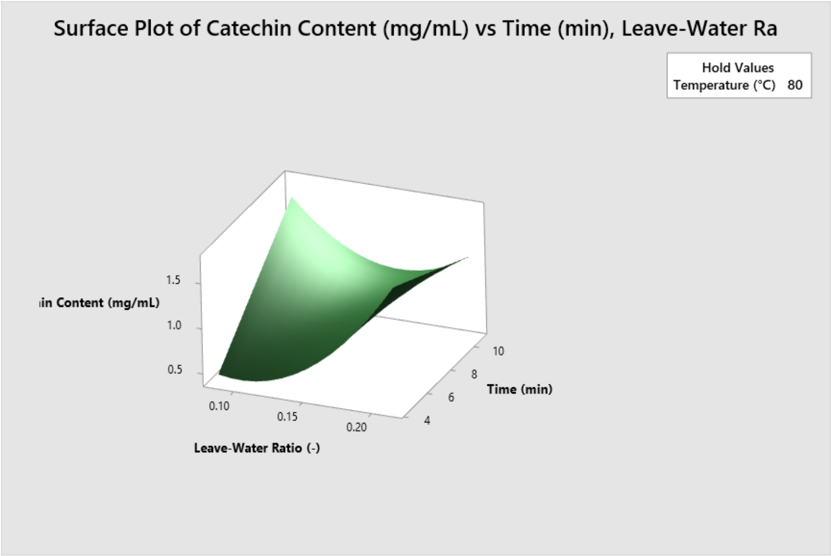

20210208,MINITAB.MWX

Response Surface Regression: Catechin Content (mg/mL) versus Temperature (°C), Leave-Water Ratio (-), Time (min)

Coded Coefficients

Term	Coef	SE Coef	T-Value	P-Value	VIF
Constant	0.8430	0.0615	13.70	0.000	
Temperature (°C)	0.2611	0.0483	5.40	0.000	1.00
Leave-Water Ratio (-)	0.1796	0.0483	3.72	0.004	1.00
Time (min)	0.1256	0.0483	2.60	0.027	1.00
Temperature (°C)*Temperature (°C)	0.5272	0.0638	8.27	0.000	1.09
Leave-Water Ratio (-)*Leave-Water Ratio (-)	0.2554	0.0638	4.01	0.002	1.09
Time (min)*Time (min)	-0.0029	0.0638	-0.05	0.964	1.09
Temperature (°C)*Leave-Water Ratio (-)	-0.0713	0.0575	-1.24	0.243	1.00
Temperature (°C)*Time (min)	-0.0964	0.0575	-1.68	0.125	1.00
Leave-Water Ratio (-)*Time (min)	-0.2456	0.0575	-4.27	0.002	1.00

Model Summary

S	R-sq	R-sq(adj)	R-sq(pred)
0.162556	94.75%	90.03%	58.94%

Analysis of Variance

Source	DF	Adj SS	Adj MS	F-Value	P-Value
Model	9	4.76947	0.52994	20.05	0.000

Linear	3	1.31473	0.43824	16.58	0.000
Temperature (°C)	1	0.77132	0.77132	29.19	0.000
Leave-Water Ratio (-)	1	0.36487	0.36487	13.81	0.004
Time (min)	1	0.17853	0.17853	6.76	0.027
Square	3	2.85735	0.95245	36.04	0.000
Temperature (°C)*Temperature (°C)	1	1.80723	1.80723	68.39	0.000
Leave-Water Ratio (-)*Leave-Water Ratio (-)	1	0.42421	0.42421	16.05	0.002
Time (min)*Time (min)	1	0.00006	0.00006	0.00	0.964
2-Way Interaction	3	0.59739	0.19913	7.54	0.006
Temperature (°C)*Leave-Water Ratio (-)	1	0.04073	0.04073	1.54	0.243
Temperature (°C)*Time (min)	1	0.07429	0.07429	2.81	0.125
Leave-Water Ratio (-)*Time (min)	1	0.48237	0.48237	18.25	0.002
Error	10	0.26425	0.02642		
Lack-of-Fit	5	0.26425	0.05285	*	*
Pure Error	5	0.00000	0.00000		
Total	19	5.03372			

Regression Equation in Uncoded Units

Catechin Content (mg/mL) = 122.7 - 3.222 Temperature (°C) + 10.5 Leave-Water Ratio (-)
+ 0.969 Time (min) + 0.02109 Temperature (°C)*Temperature (°C)
+ 102.2 Leave-Water Ratio (-)*Leave-Water Ratio (-)
- 0.0005 Time (min)*Time (min)
- 0.285 Temperature (°C)*Leave-Water Ratio (-)
- 0.00771 Temperature (°C)*Time (min)
- 1.964 Leave-Water Ratio (-)*Time (min)

Fits and Diagnostics for Unusual Observations

Obs	Catechin Content (mg/mL)	Fit	Resid	Std Resid
10	2.279	2.053	0.226	2.03 R

R Large residual

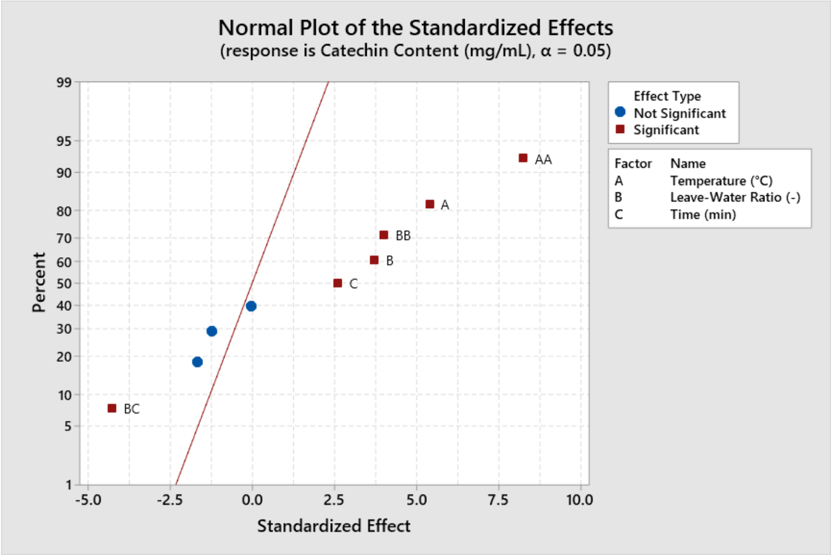

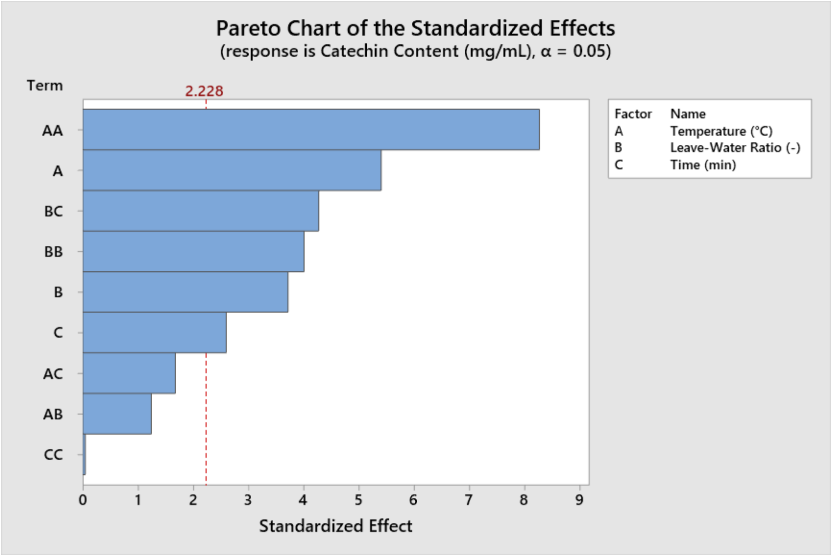

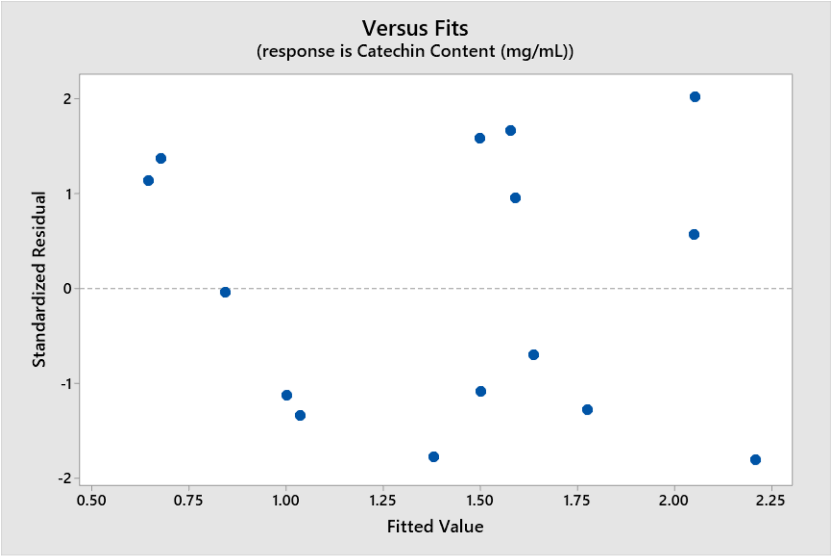

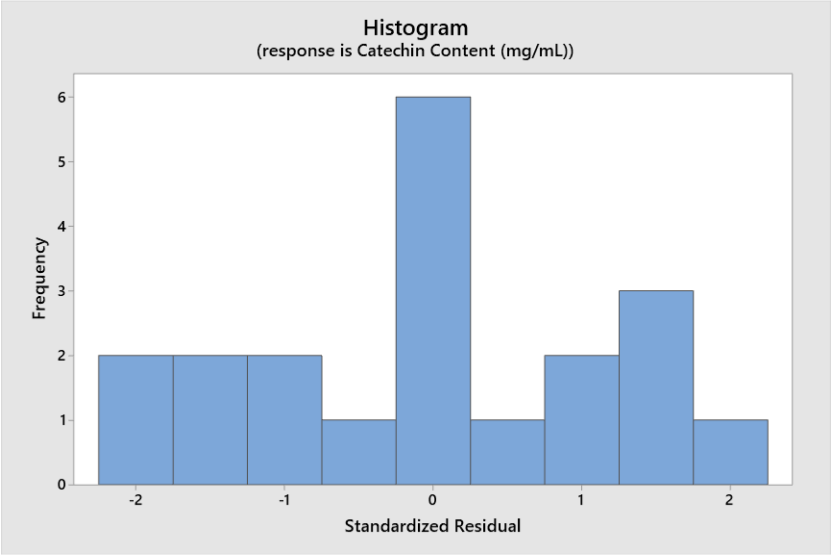

20210208,MINITAB.MWX

Contour Plots of Caffeine Content (mg/mL)

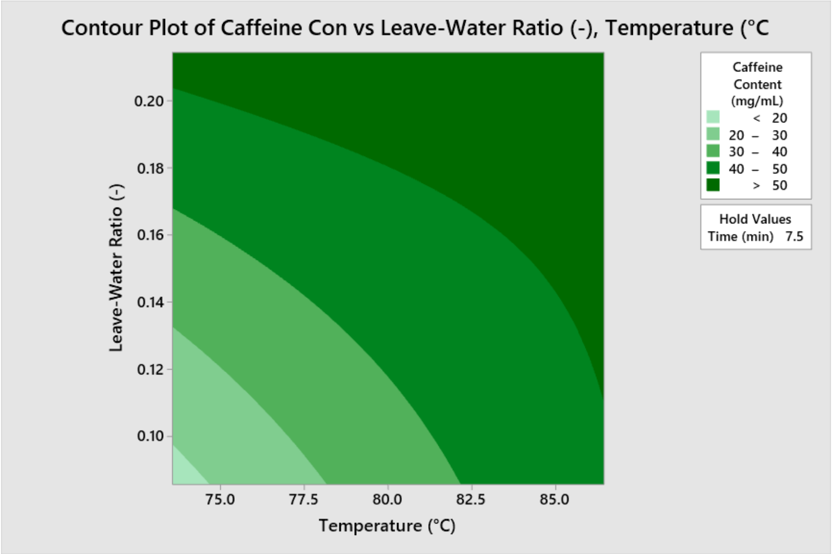

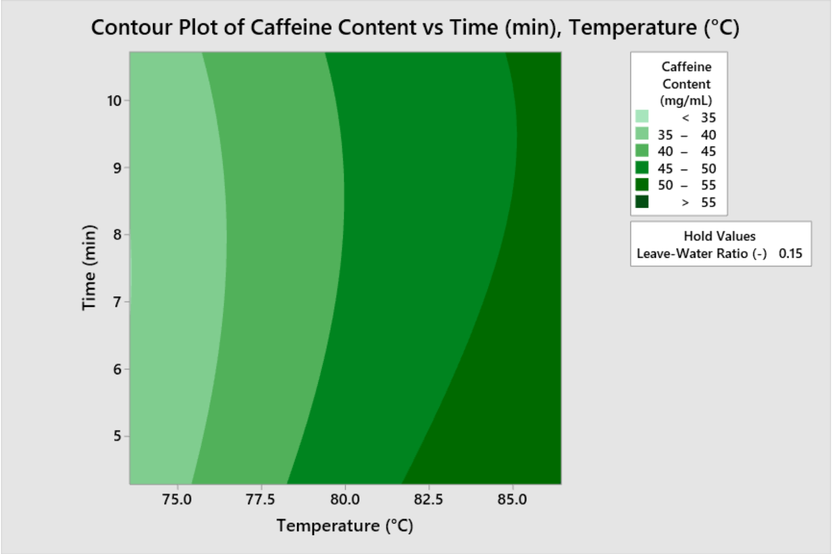

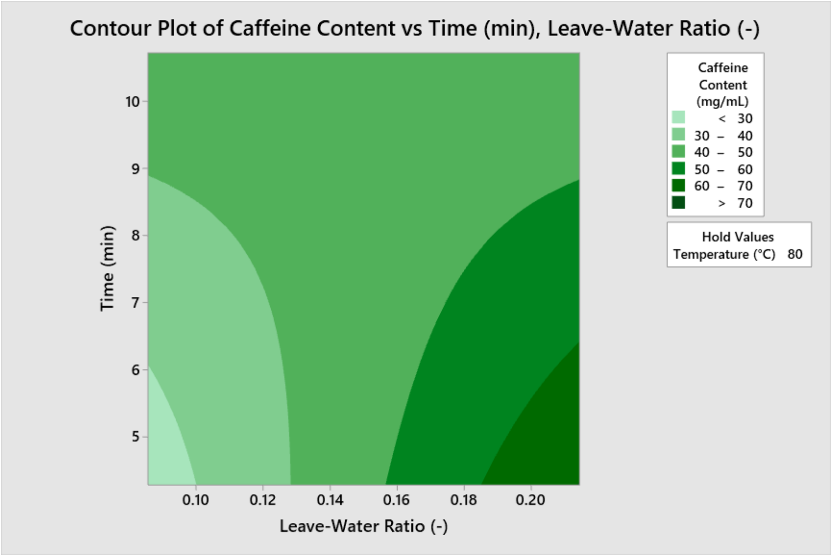

20210208,MINITAB.MWX

Surface Plots of Caffeine Content (mg/mL)

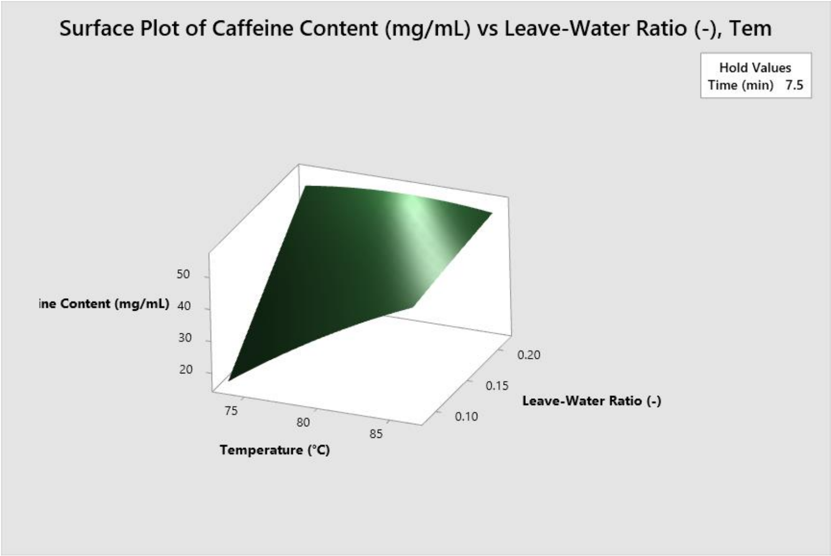

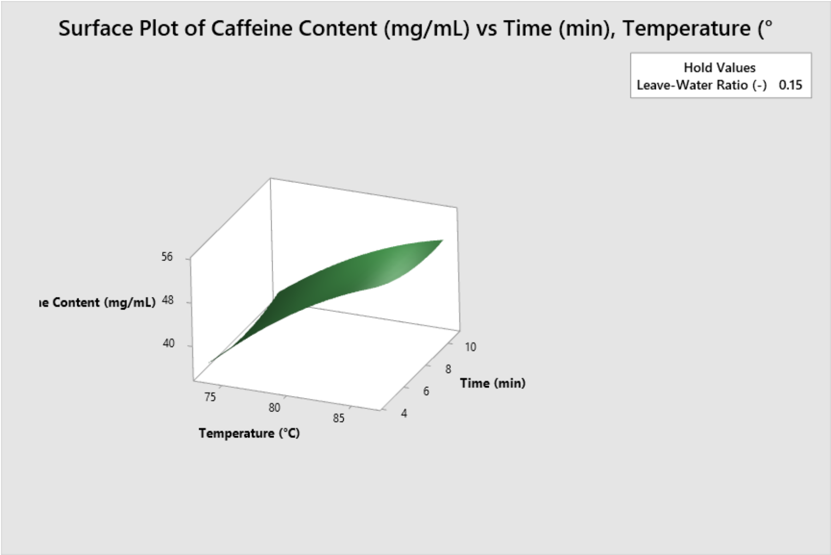

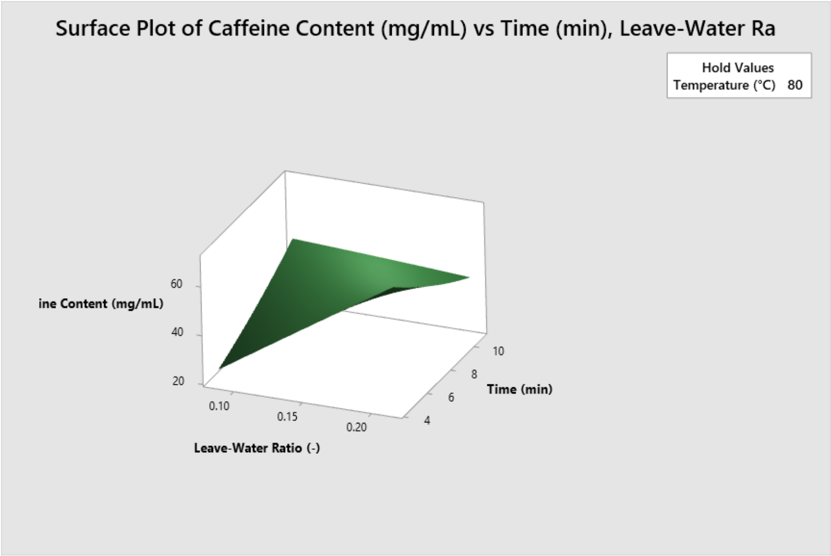

20210208,MINITAB.MWX

Response Surface Regression: Caffeine Content (mg/mL) versus Temperature (°C), Leave-Water Ratio (-), Time (min)

Coded Coefficients

Term	Coef	SE Coef	T-Value	P-Value	VIF
Constant	45.20	2.00	22.66	0.000	
Temperature (°C)	6.46	1.57	4.12	0.002	1.00
Leave-Water Ratio (-)	7.97	1.57	5.09	0.000	1.00
Time (min)	-0.77	1.57	-0.49	0.633	1.00
Temperature (°C)*Temperature (°C)	-1.20	2.07	-0.58	0.574	1.09
Leave-Water Ratio (-)*Leave-Water Ratio (-)	-0.10	2.07	-0.05	0.961	1.09
Time (min)*Time (min)	0.91	2.07	0.44	0.670	1.09
Temperature (°C)*Leave-Water Ratio (-)	-4.79	1.86	-2.57	0.028	1.00
Temperature (°C)*Time (min)	-0.64	1.86	-0.34	0.740	1.00
Leave-Water Ratio (-)*Time (min)	-7.50	1.86	-4.02	0.002	1.00

Model Summary

S	R-sq	R-sq(adj)	R-sq(pred)
5.27095	86.92%	75.15%	0.00%

Analysis of Variance

Source	DF	Adj SS	Adj MS	F-Value	P-Value
Model	9	1846.76	205.195	7.39	0.002

Linear	3	1197.40	399.135	14.37	0.001
Temperature (°C)	1	471.61	471.612	16.97	0.002
Leave-Water Ratio (-)	1	719.07	719.066	25.88	0.000
Time (min)	1	6.73	6.726	0.24	0.633
Square	3	12.73	4.244	0.15	0.926
Temperature (°C)*Temperature (°C)	1	9.36	9.360	0.34	0.574
Leave-Water Ratio (-)*Leave-Water Ratio (-)	1	0.07	0.070	0.00	0.961
Time (min)*Time (min)	1	5.35	5.346	0.19	0.670
2-Way Interaction	3	636.62	212.208	7.64	0.006
Temperature (°C)*Leave-Water Ratio (-)	1	183.38	183.379	6.60	0.028
Temperature (°C)*Time (min)	1	3.24	3.244	0.12	0.740
Leave-Water Ratio (-)*Time (min)	1	450.00	450.000	16.20	0.002
Error	10	277.83	27.783		
Lack-of-Fit	5	277.83	55.566	*	*
Pure Error	5	0.00	0.000		
Total	19	2124.59			

Regression Equation in Uncoded Units

Caffeine Content (mg/mL) = -708 + 12.2 Temperature (°C) + 2154 Leave-Water Ratio (-)
+ 10.6 Time (min) - 0.0480 Temperature (°C)*Temperature (°C)
- 42 Leave-Water Ratio (-)*Leave-Water Ratio (-)
+ 0.145 Time (min)*Time (min)
- 19.15 Temperature (°C)*Leave-Water Ratio (-)
- 0.051 Temperature (°C)*Time (min)
- 60.0 Leave-Water Ratio (-)*Time (min)

Fits and Diagnostics for Unusual Observations

Obs	Caffeine Content (mg/mL)	Fit	Resid	Std Resid	
1	24.17	18.22	5.95	2.23	R
2	36.58	41.99	-5.41	-2.03	R
8	37.94	45.54	-7.60	-2.85	R
10	58.89	51.53	7.36	2.04	R

R Large residual

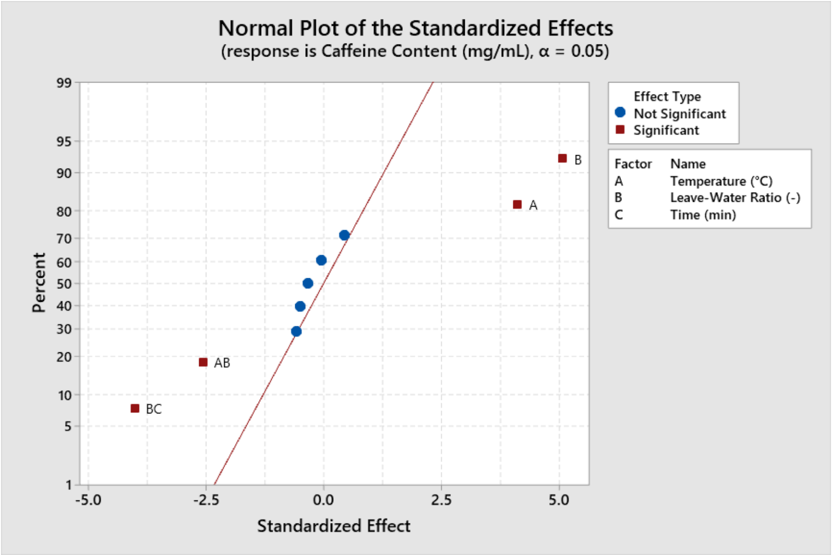

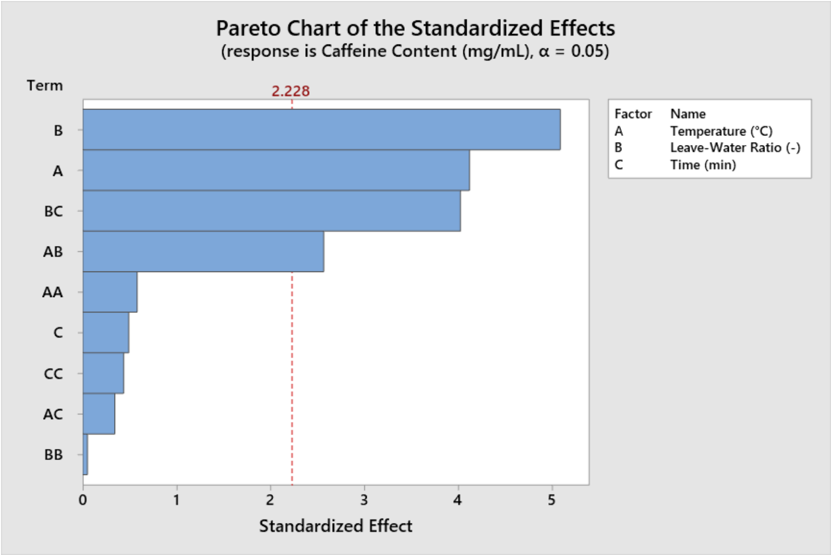
Pareto Chart of the Standardized Effects
(response is Caffeine Content (mg/mL), α = 0.05)
Term
2.228
B
A
BC
AB
AA
C
CC
AC
BB
0
1
2
3
4
5
Standardized Effect
Factor Name
A Temperature (°C)
B Leave-Water Ratio (-)
C Time (min)

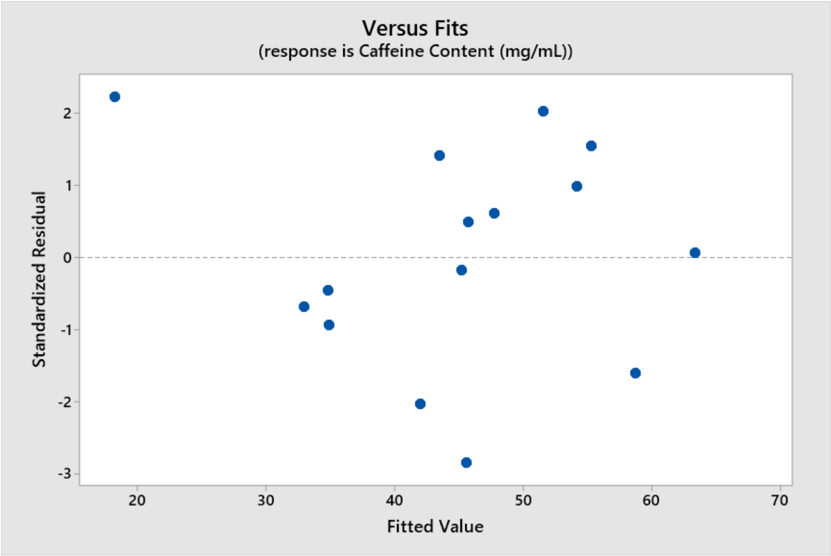
Versus Fits
(response is Caffeine Content (mg/mL))
Standardized Residual
2
1
0
-1
-2
-3
20
30
40
50
60
70
Fitted Value

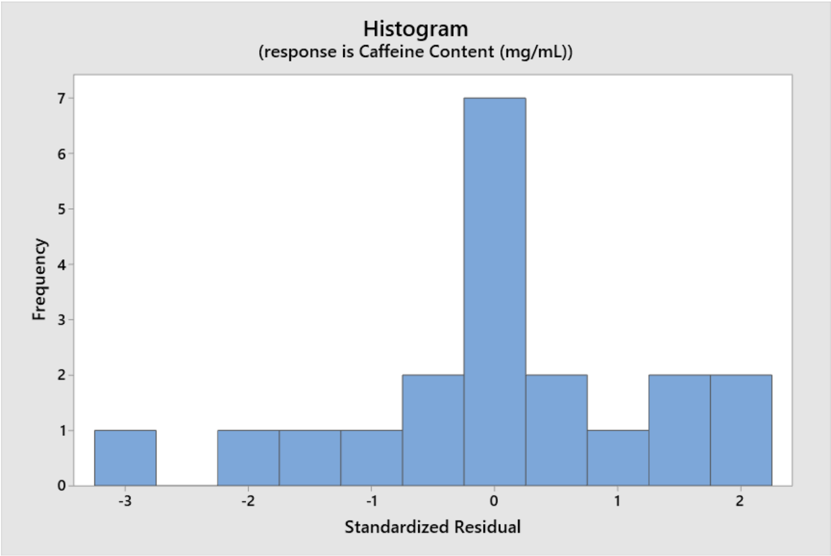
Histogram
(response is Caffeine Content (mg/mL))
Frequency
7
6
5
4
3
2
1
0
-3
-2
-1
0
1
2
Standardized Residual

Contour Plots of Phenol Content (mg/g)

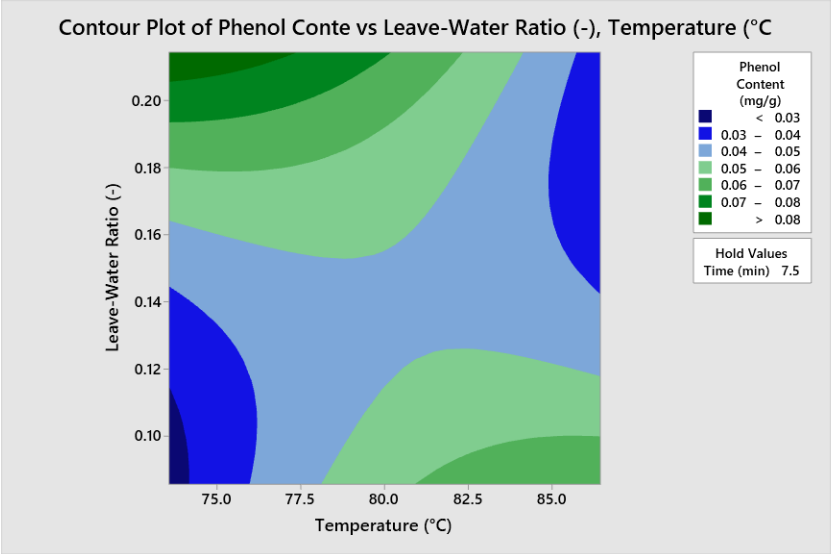

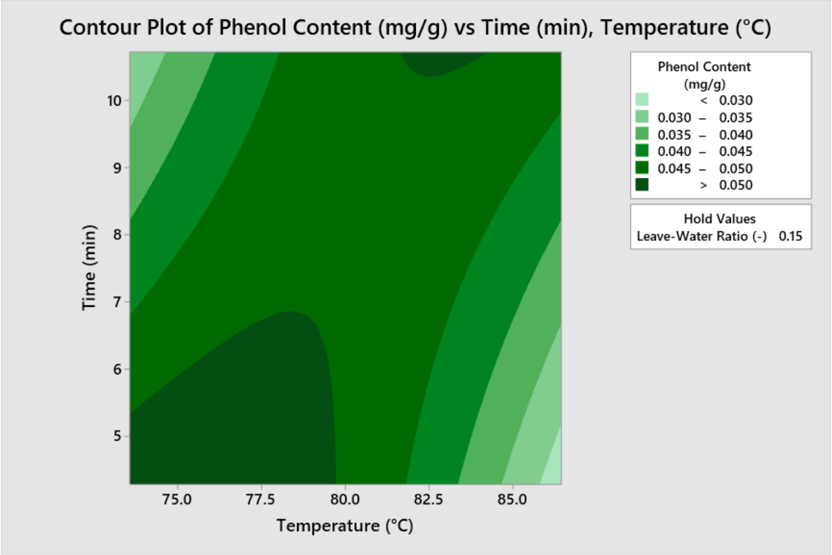

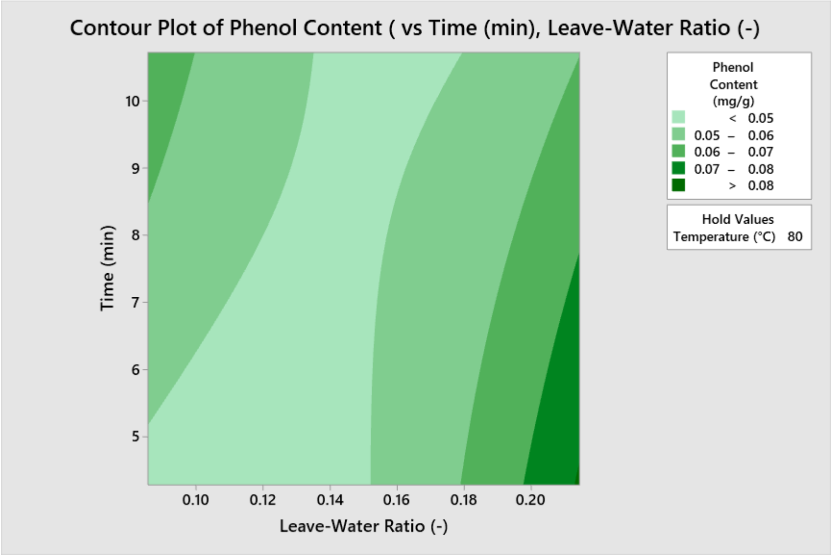

20210208,MINITAB.MWX

Surface Plots of Phenol Content (mg/g)

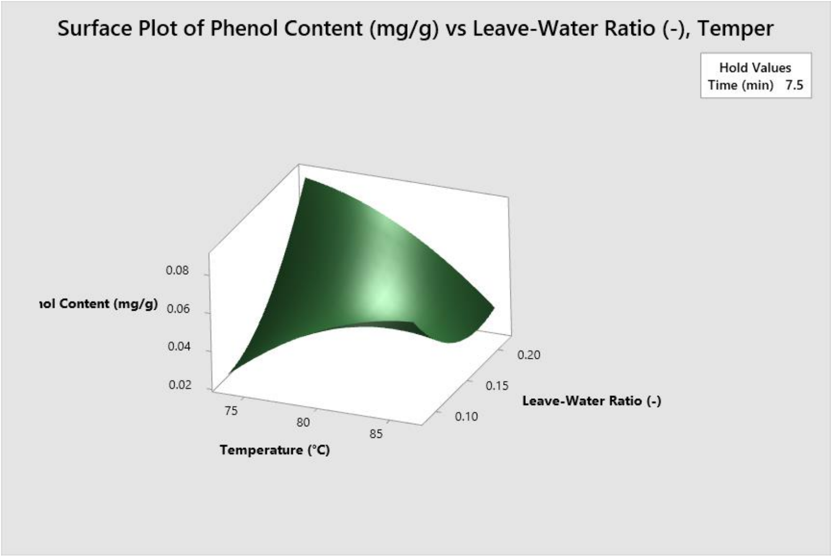

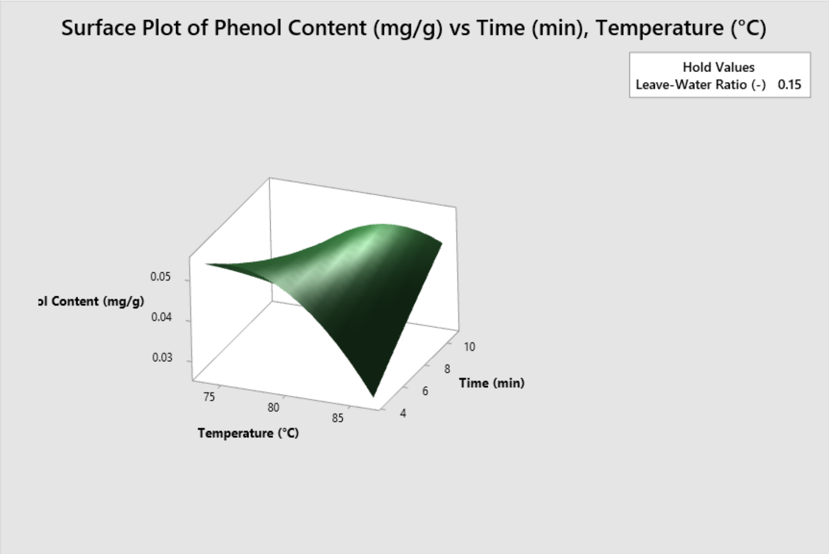

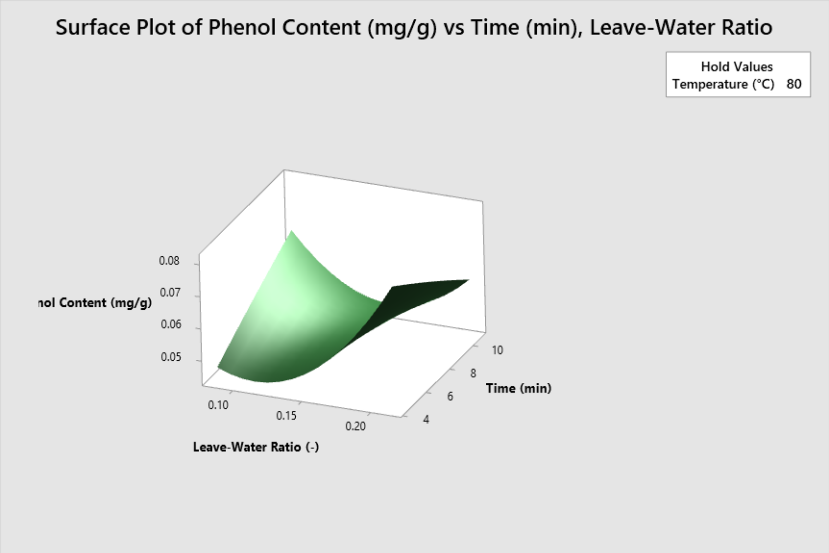

20210208,MINITAB.MWX

Response Surface Regression: Phenol Content (mg/g) versus Temperature (°C), Leave-Water Ratio (-), Time (min)

Coded Coefficients

Term	Coef	SE Coef	T-Value	P-Value	VIF
Constant	0.04936	0.00560	8.81	0.000	
Temperature (°C)	-0.00187	0.00440	-0.42	0.680	1.00
Leave-Water Ratio (-)	0.00529	0.00440	1.20	0.257	1.00
Time (min)	-0.00039	0.00440	-0.09	0.932	1.00
Temperature (°C)*Temperature (°C)	-0.00557	0.00580	-0.96	0.360	1.09
Leave-Water Ratio (-)*Leave-Water Ratio (-)	0.00883	0.00580	1.52	0.159	1.09
Time (min)*Time (min)	-0.00025	0.00580	-0.04	0.967	1.09
Temperature (°C)*Leave-Water Ratio (-)	-0.01448	0.00523	-2.77	0.020	1.00
Temperature (°C)*Time (min)	0.00656	0.00523	1.25	0.239	1.00
Leave-Water Ratio (-)*Time (min)	-0.00611	0.00523	-1.17	0.270	1.00

Model Summary

S	R-sq	R-sq(adj)	R-sq(pred)
0.0147935	60.07%	24.13%	0.00%

Analysis of Variance

Source	DF	Adj SS	Adj MS	F-Value	P-Value
Model	9	0.003292	0.000366	1.67	0.217

Linear	3	0.000358	0.000119	0.55	0.662
Temperature (°C)	1	0.000039	0.000039	0.18	0.680
Leave-Water Ratio (-)	1	0.000317	0.000317	1.45	0.257
Time (min)	1	0.000002	0.000002	0.01	0.932
Square	3	0.000614	0.000205	0.93	0.460
Temperature (°C)*Temperature (°C)	1	0.000202	0.000202	0.92	0.360
Leave-Water Ratio (-)*Leave-Water Ratio (-)	1	0.000507	0.000507	2.32	0.159
Time (min)*Time (min)	1	0.000000	0.000000	0.00	0.967
2-Way Interaction	3	0.002320	0.000773	3.53	0.056
Temperature (°C)*Leave-Water Ratio (-)	1	0.001678	0.001678	7.67	0.020
Temperature (°C)*Time (min)	1	0.000344	0.000344	1.57	0.239
Leave-Water Ratio (-)*Time (min)	1	0.000299	0.000299	1.36	0.270
Error	10	0.002188	0.000219		
Lack-of-Fit	5	0.002188	0.000438	*	*
Pure Error	5	0.000000	0.000000		
Total	19	0.005480			

Regression Equation in Uncoded Units

Phenol Content (mg/g) = -1.72 + 0.0400 Temperature (°C) + 4.05 Leave-Water Ratio (-)
- 0.0342 Time (min) - 0.000223 Temperature (°C)*Temperature (°C)
+ 3.53 Leave-Water Ratio (-)*Leave-Water Ratio (-)
- 0.000040 Time (min)*Time (min)
- 0.0579 Temperature (°C)*Leave-Water Ratio (-)
+ 0.000525 Temperature (°C)*Time (min)
- 0.0489 Leave-Water Ratio (-)*Time (min)

Fits and Diagnostics for Unusual Observations

Obs	Phenol Content (mg/g)	Fit	Resid	Std Resid
4	0.02423	0.04125	-0.01703	-2.28 R
5	0.05234	0.03363	0.01871	2.51 R

R Large residual

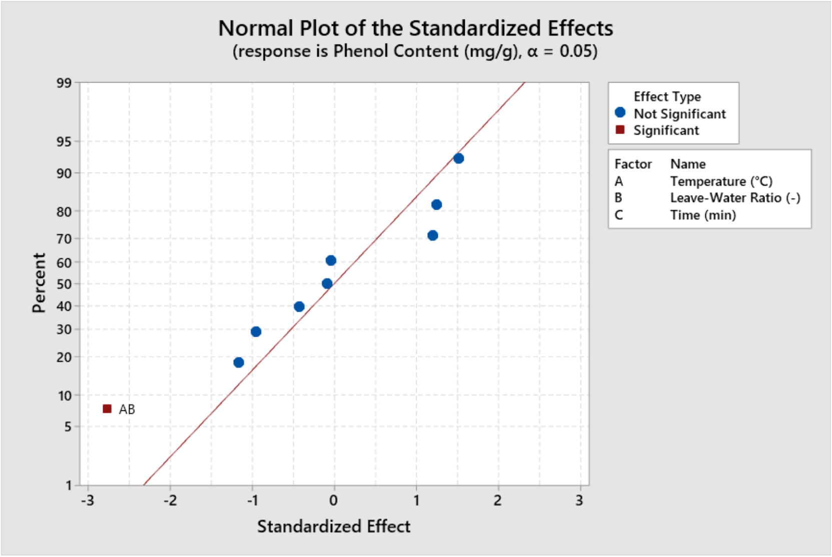

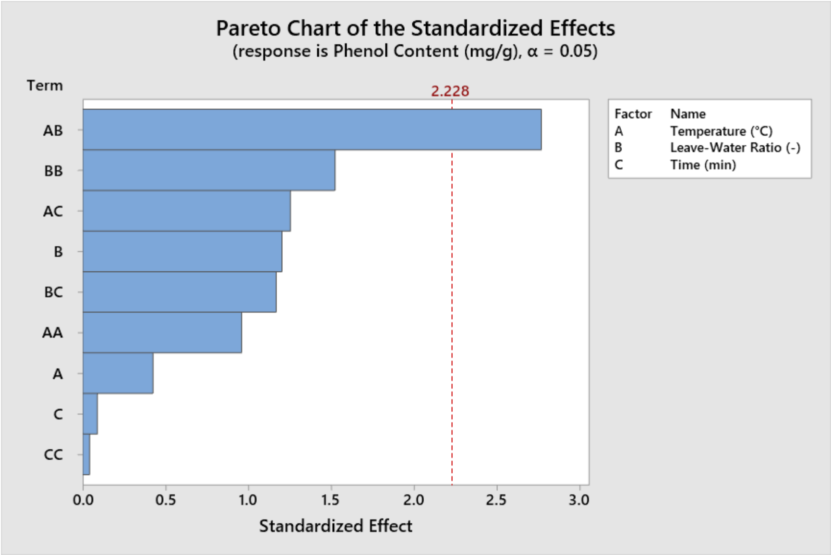

Pareto Chart of the Standardized Effects
(response is Phenol Content (mg/g), α = 0.05)
Term
2.228
AB
BB
AC
B
BC
AA
A
C
CC
0.0
0.5
1.0
1.5
2.0
2.5
3.0
Standardized Effect
Factor Name
A Temperature (°C)
B Leave-Water Ratio (-)
C Time (min)

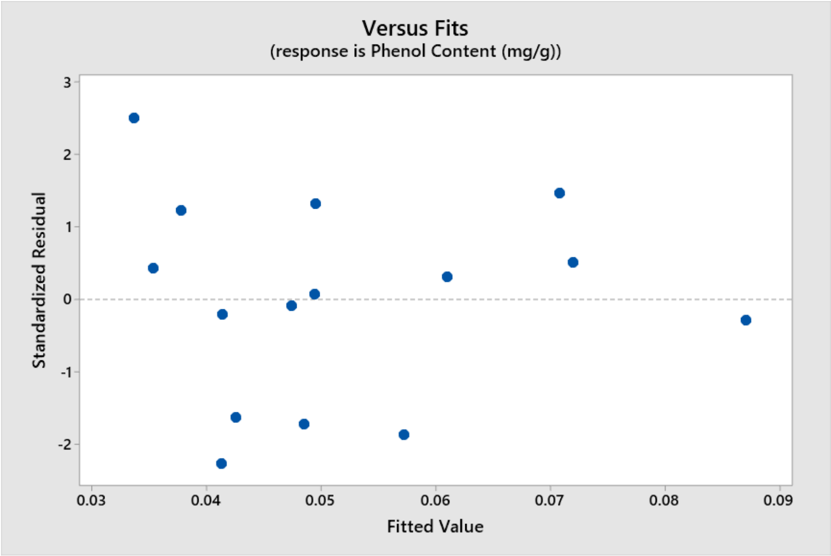

Versus Fits
(response is Phenol Content (mg/g))
Standardized Residual
3
2
1
0
-1
-2
0.03
0.04
0.05
0.06
0.07
0.08
0.09
Fitted Value

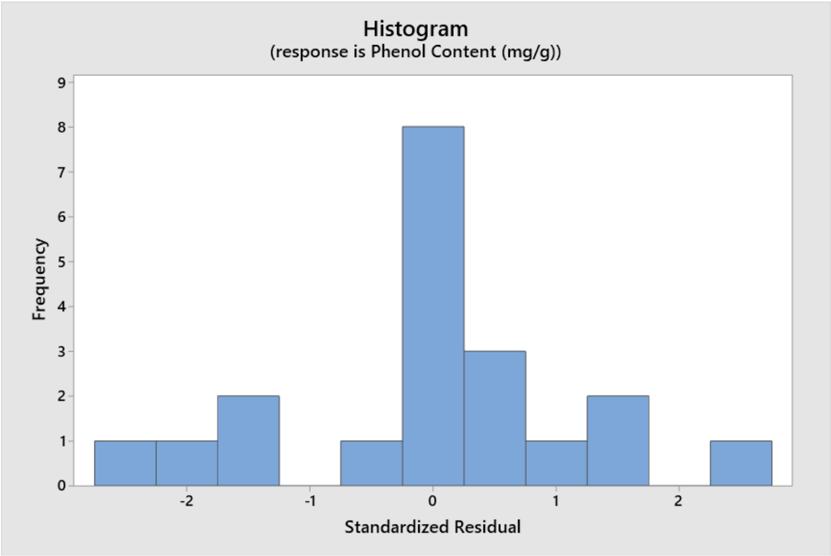

Histogram
(response is Phenol Content (mg/g))
Frequency
9
8
7
6
5
4
3
2
1
0
-2
-1
0
1
2
Standardized Residual

20210208,MINITAB.MWX

Contour Plots of Powder Particle Size (micro-m)

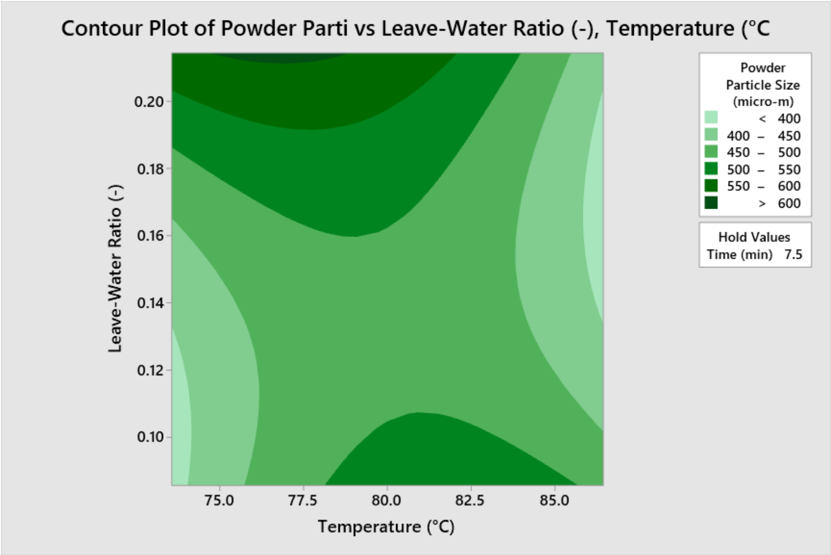

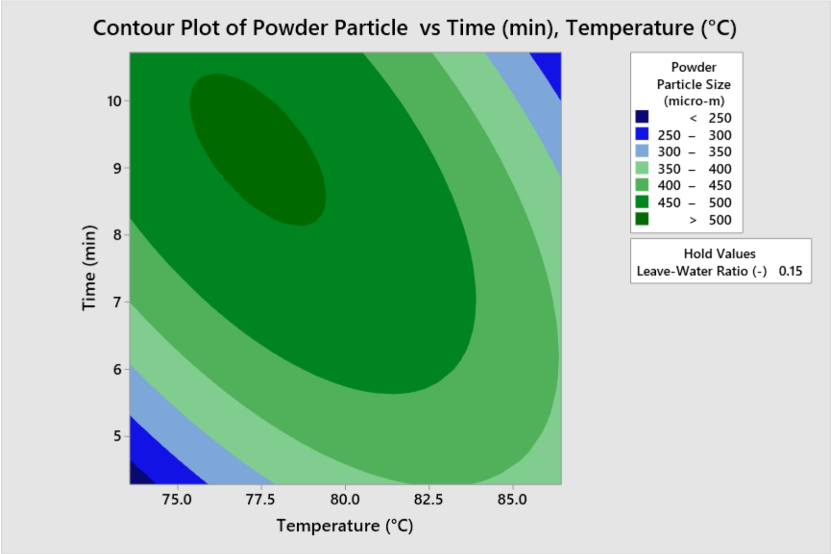

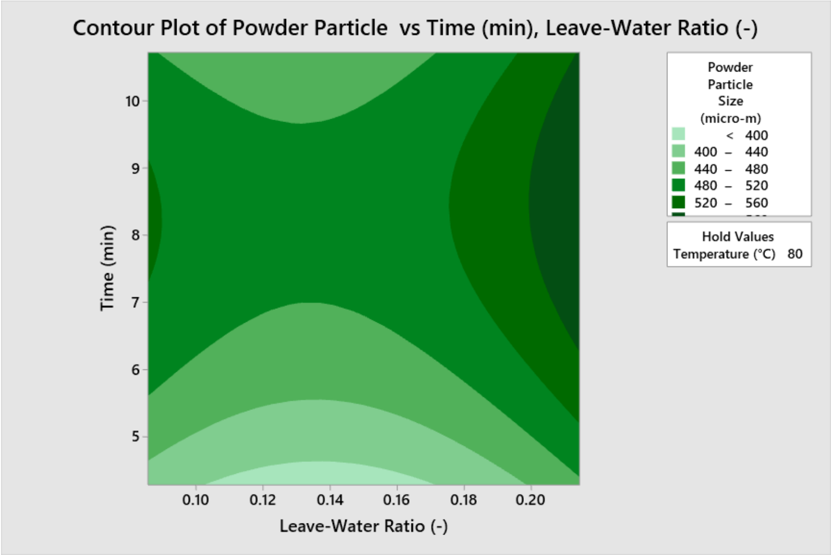

20210208,MINITAB.MWX

Surface Plots of Powder Particle Size (micro-m)

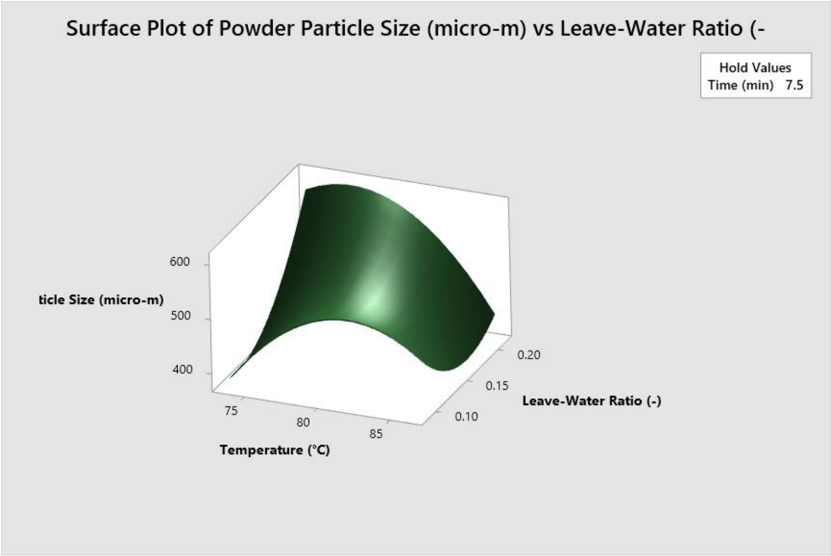

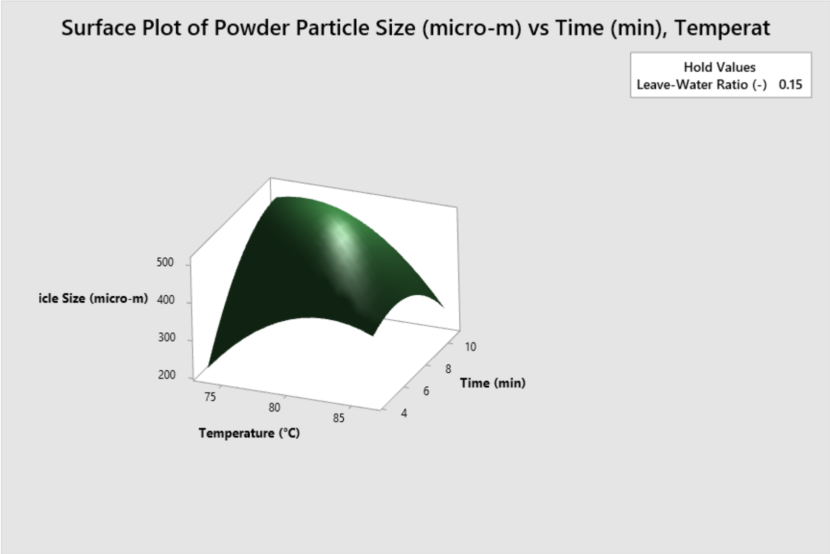

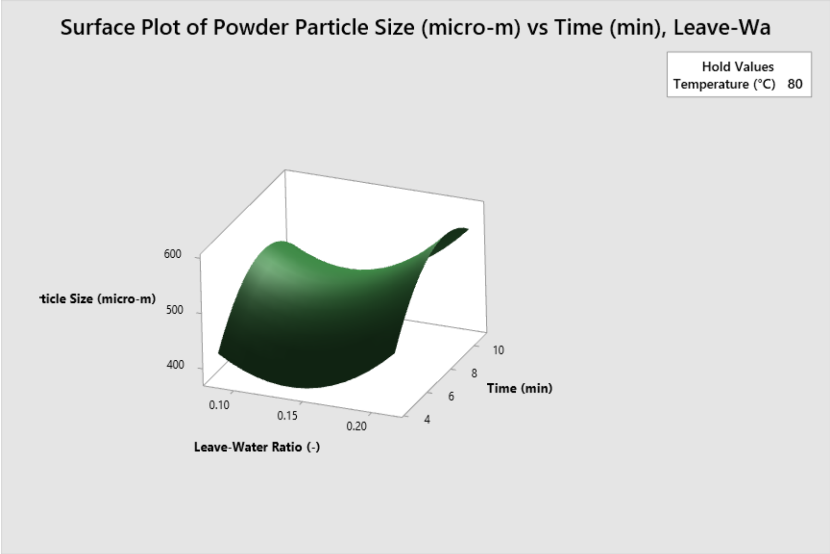

20210208,MINITAB.MWX

Response Surface Regression: Powder Particle Size (micro-m) versus Temperature (°C), Leave-Water Ratio (-), Time (min)

Coded Coefficients

Term	Coef	SE Coef	T-Value	P-Value	VIF
Constant	491.4	36.2	13.59	0.000	
Temperature (°C)	-14.1	28.4	-0.50	0.631	1.00
Leave-Water Ratio (-)	25.2	28.4	0.89	0.396	1.00
Time (min)	29.2	28.4	1.03	0.328	1.00
Temperature (°C)*Temperature (°C)	-52.5	37.5	-1.40	0.192	1.09
Leave-Water Ratio (-)*Leave-Water Ratio (-)	38.3	37.5	1.02	0.331	1.09
Time (min)*Time (min)	-41.9	37.5	-1.12	0.289	1.09
Temperature (°C)*Leave-Water Ratio (-)	-41.9	33.8	-1.24	0.243	1.00
Temperature (°C)*Time (min)	-57.6	33.8	-1.71	0.119	1.00
Leave-Water Ratio (-)*Time (min)	4.1	33.8	0.12	0.906	1.00

Model Summary

S	R-sq	R-sq(adj)	R-sq(pred)
95.5433	51.69%	8.21%	0.00%

Analysis of Variance

Source	DF	Adj SS	Adj MS	F-Value	P-Value
Model	9	97661	10851.3	1.19	0.393

	DF	Adj SS	Adj MS	F-Value	P-Value
Linear	3	19074	6358.0	0.70	0.575
Temperature (°C)	1	2238	2238.0	0.25	0.631
Leave-Water Ratio (-)	1	7190	7189.7	0.79	0.396
Time (min)	1	9646	9646.1	1.06	0.328
Square	3	37860	12620.0	1.38	0.304
Temperature (°C)*Temperature (°C)	1	17902	17902.5	1.96	0.192
Leave-Water Ratio (-)*Leave-Water Ratio (-)	1	9522	9521.6	1.04	0.331
Time (min)*Time (min)	1	11434	11434.3	1.25	0.289
2-Way Interaction	3	40728	13575.8	1.49	0.277
Temperature (°C)*Leave-Water Ratio (-)	1	14030	14029.6	1.54	0.243
Temperature (°C)*Time (min)	1	26565	26565.0	2.91	0.119
Leave-Water Ratio (-)*Time (min)	1	133	132.9	0.01	0.906
Error	10	91285	9128.5		
Lack-of-Fit	5	91285	18257.1	*	*
Pure Error	5	0	0.0		
Total	19	188947			

Regression Equation in Uncoded Units

Powder Particle Size (micro-m) = -17653 + 393 Temperature (°C) + 9068 Leave-Water Ratio (-
)
+ 476 Time (min) - 2.10 Temperature (°C)*Temperature (°C)
+ 15308 Leave-Water Ratio (-)*Leave-Water Ratio (-)
- 6.71 Time (min)*Time (min)
- 168 Temperature (°C)*Leave-Water Ratio (-)
- 4.61 Temperature (°C)*Time (min)
+ 33 Leave-Water Ratio (-)*Time (min)

Fits and Diagnostics for Unusual Observations

Obs	Powder Particle Size (micro-m)	Fit	Resid	Std Resid	
5	564.5	465.0	99.5	2.06	R
11	353.2	522.4	-169.2	-2.58	R
12	738.4	587.3	151.1	2.31	R

R Large residual

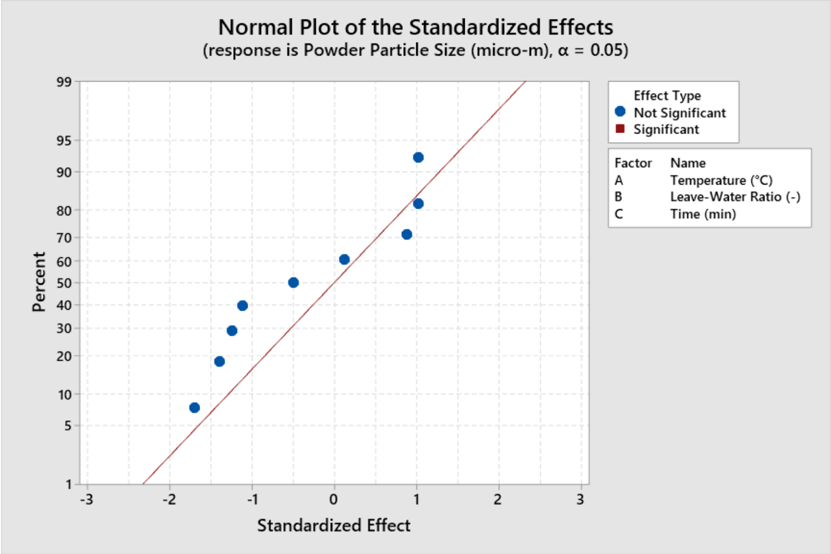

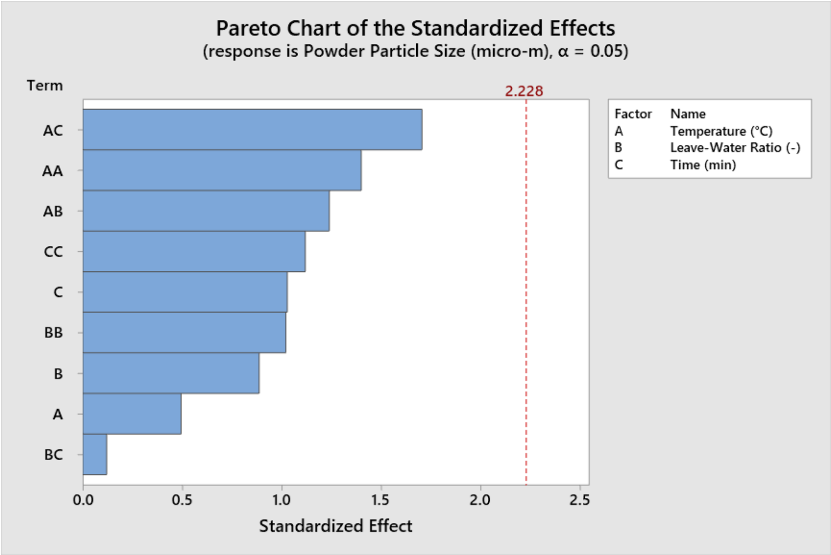
Pareto Chart of the Standardized Effects
(response is Powder Particle Size (micro-m), α = 0.05)
Term
2.228
AC
AA
AB
CC
C
BB
B
A
BC
0.0
0.5
1.0
1.5
2.0
2.5
Standardized Effect
Factor Name
A Temperature (°C)
B Leave-Water Ratio (-)
C Time (min)

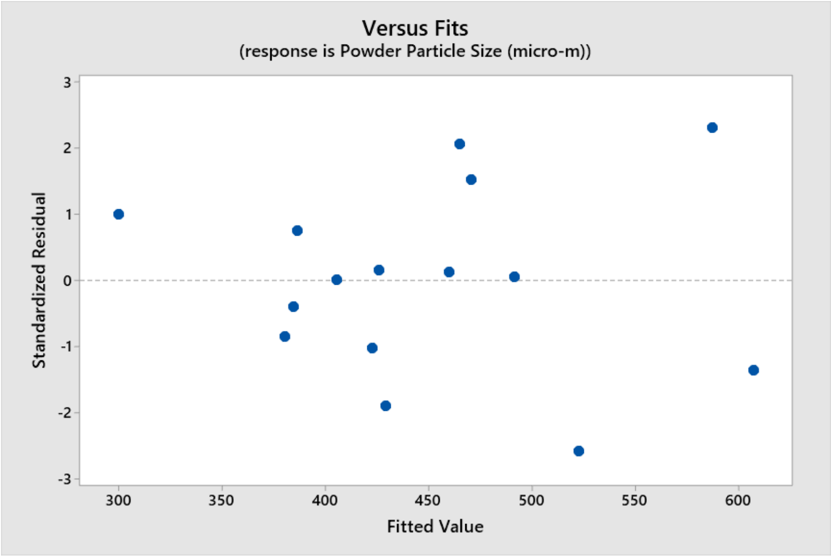
Versus Fits
(response is Powder Particle Size (micro-m))
Standardized Residual
3
2
1
0
-1
-2
-3
300
350
400
450
500
550
600
Fitted Value

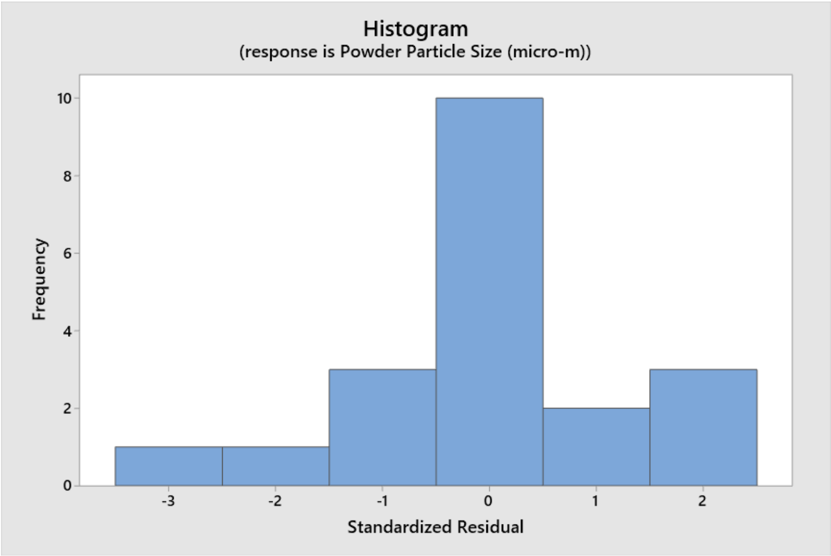
Histogram
(response is Powder Particle Size (micro-m))
Frequency
10
8
6
4
2
0
-3
-2
-1
0
1
2
Standardized Residual
